# Decrypting bacterial polyphenol metabolism in an anoxic wetland soil

**DOI:** 10.1038/s41467-021-22765-1

**Published:** 2021-04-29

**Authors:** Bridget B. McGivern, Malak M. Tfaily, Mikayla A. Borton, Suzanne M. Kosina, Rebecca A. Daly, Carrie D. Nicora, Samuel O. Purvine, Allison R. Wong, Mary S. Lipton, David W. Hoyt, Trent R. Northen, Ann E. Hagerman, Kelly C. Wrighton

**Affiliations:** 1 Colorado State University, Fort Collins, CO USA; 2 University of Arizona, Tucson, AZ USA; 3 Environmental Genomics and Systems Biology, Lawrence Berkeley National Laboratory, Berkeley, CA USA; 4 Pacific Northwest National Laboratory, Richland, WA USA; 5 Miami University, Oxford, OH USA

**Keywords:** Soil microbiology, Carbon cycle

## Abstract

Microorganisms play vital roles in modulating organic matter decomposition and nutrient cycling in soil ecosystems. The enzyme latch paradigm posits microbial degradation of polyphenols is hindered in anoxic peat leading to polyphenol accumulation, and consequently diminished microbial activity. This model assumes that polyphenols are microbially unavailable under anoxia, a supposition that has not been thoroughly investigated in any soil type. Here, we use anoxic soil reactors amended with and without a chemically defined polyphenol to test this hypothesis, employing metabolomics and genome-resolved metaproteomics to interrogate soil microbial polyphenol metabolism. Challenging the idea that polyphenols are not bioavailable under anoxia, we provide metabolite evidence that polyphenols are depolymerized, resulting in monomer accumulation, followed by the generation of small phenolic degradation products. Further, we show that soil microbiome function is maintained, and possibly enhanced, with polyphenol addition. In summary, this study provides chemical and enzymatic evidence that some soil microbiota can degrade polyphenols under anoxia and subvert the assumed polyphenol lock on soil microbial metabolism.

## Abstract (web summary)

It is thought that polyphenols inhibit organic matter decomposition in soils devoid of oxygen. Here the authors use metabolomics and genome-resolved metaproteomics to provide experimental evidence of polyphenol biodegradation and maintained soil microbial community metabolism despite anoxia.

## Introduction

Polyphenols are one of the most abundant types of plant secondary metabolites. This prevalent chemical group is heterogeneous, consisting of over 10,000 structurally divergent compounds^1^. These compounds are abundant in differing habitats: they enter the soil systems through litter decay or leaching^2^, while in gut systems these plant-derived metabolites are consumed in high concentrations from polyphenol-rich foods like berries and cocoa^3^. In the human gut, it is recognized that the gut microbiome plays an integral role in the anaerobic processing of dietary polyphenols to enable host absorption^3^. Similarly, in ruminants, microbial interactions with polyphenols in feed have ramifications for animal nutrition and husbandry^4^. Despite the prevalence and recognized importance of polyphenol compounds, the mechanisms underlying anaerobic microbial polyphenol metabolism are just being unveiled in gut systems^5,6^, and remain largely enigmatic in soil systems.

**Fig. 1:**
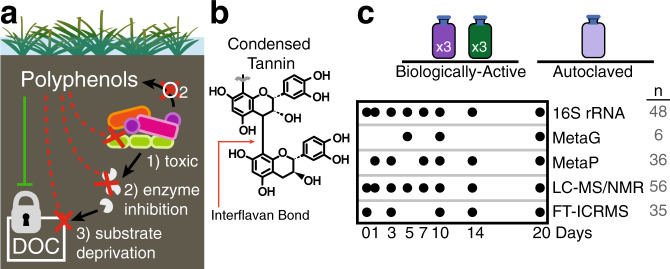
Experimental framework using a model polyphenol to interrogate soil microbial metabolism. **a** Schematic summarizing the polyphenol lock paradigm^9,10^, demonstrating the ways these compounds may control microbial carbon transformations in anoxic soils. The green solid line indicates that in anoxic soils, polyphenols promote the lock on dissolved organic carbon (DOC). The dotted-red lines show the three proposed mechanisms by which polyphenols restrict the activity of soil microorganisms to lock soil carbon, including (1) toxicity to microorganisms, (2) inhibiting microbial extracellular enzymes, and (3) binding and depriving microorganisms of nutrients. **b** A purified condensed tannin (CT) was selected as the model polyphenol in this study due to its inferred lack of microbial degradation in anoxic soils^7^. This model compound is well characterized chemically^21^ and has an average degree of polymerization of 16, where interflavan bonds (red arrow) connect monomers of epicatechin with a single catechin monomer cap. **c** The experimental design included soil reactors from three treatments (i) Biologically-active CT amended (dark purple), (ii) Biologically-active unamended control (green), and (iii) CT-amended autoclaved control (light purple). Autoclaved soils only included metabolite analyses, while microbially-active soils were analyzed with the suite of multi-omics approaches. The timepoints of each type of analysis are shown, with the total number (n) of samples across treatments denoted on the right in gray.

Despite knowledge from gut systems, in soils, and especially in polyphenol-rich peatlands^7^, it is widely assumed that microbial polyphenol degradation is an obligately aerobic metabolism, and thus cannot occur under anoxia. Consequently, the “enzyme latch”^8,9^ hypothesis states that polyphenols accumulate under anoxic soil conditions and further control soil microbial carbon cycling as these compounds (1) are toxic to microorganisms, (2) inactivate microbial extracellular enzymes, and/or (3) bind substrates, thus depriving microorganisms of nutrients and limiting microbial activity^10^ (Fig. 1a). According to this model, polyphenols serve as a “lock”^10^ to stabilize soil carbon in anoxic soils (Fig. 1a). Based on these assumptions, it has been proposed that polyphenol amendment can be a tool for slowing rates of soil organic matter decomposition to mitigate carbon loss from peatlands^7–9,11^. However, the studies supporting these assertions in peat, or in any soil system, have not directly interrogated microbial metabolism in anoxic soils, instead inferring microbial community activity from bulk level properties like respiration rate^12–14^, enzyme assays^7–9^, inferred biomass^12,15^, or cellular morphology^10^. These poorly defined interactions between soil microbiota and polyphenols must be elucidated to resolve the role of these compounds in soil carbon sequestration, especially in the face of changing climate.

Recent developments in genome sequencing technology coupled to improved computational methods make historically complex soil communities more tractable with multiple ‘omics approaches^16–18^. These methodological advances afford a renewed opportunity to discover the biochemistry underpinning microbial-polyphenol responses in soils^16,17^. Here our research goals include (i) investigating the possibility of microbially-mediated polyphenol transformations in anoxic soils, and (ii) determining the impact of polyphenols on overall microbial community function. To resolve these fates of polyphenols in anoxic soils, we use a structurally defined, model polyphenol substrate—a condensed tannin—as an amendment to our controlled, anoxic soil reactors. Periodically over 20 days, we probe this model soil microbiome with a variety of metabolomic methods and genome-resolved metaproteomics to discern the biotic and abiotic responses to the model polyphenol under anoxia. Collectively, our findings provide multi-omics evidence for polyphenol degradation and maintenance of overall microbial community function. These results represent a critical step in describing microbial polyphenol metabolism in an anoxic soil, refining the presumed metabolic roles of soil microbiota in long-held soil biogeochemical paradigms.

## Results

### Establishing laboratory microcosms to explore polyphenol fate in anoxic soils

To date many studies of the effects of polyphenols on soil microbiota have focused on boreal peat soils^7,12^. Yet in these soils, temperature (<20 °C) was suggested to be a possible kinetic controller on microbial growth and enzyme activity, thus limiting polyphenol metabolism^19^. To extend these prior studies, we selected plant-covered, mineral soils from a microbially well-studied temperate, freshwater wetland^18,20^, thereby eliminating kinetic constraints and expanding our search for these metabolisms across a broader range of soil types. These wetland surface soils contained polyphenols (Supplementary Fig. 1) and have been shown to be tractable using multi-omics methods^18,20^, and thus were used as a model soil for evaluation of anaerobic polyphenol metabolism.

Using these surface soils as the inoculum, we amended anoxic laboratory microcosms with and without a model polyphenol. Owing to the known chemical heterogeneity among polyphenols, a structurally-characterized condensed tannin^21^ (CT, Fig. 1b) was selected as a model polyphenol substrate. CT are generally recognized as recalcitrant in diverse soils^22,23^, and were recently described as a significant inhibitor of microbial activity in a riparian peatland^7^. The CT polymer is comprised of oligomers of epicatechin with a terminal catechin unit, all of which are connected by interflavan bonds (Fig. 1b). The average degree of polymerization is 16, yielding an average molecular weight of 4600 Da^21^. Reactors were amended with a CT loading of 375 mg CT/g soil, which is on par with reports of polyphenols in soils (up to 100 mg/g soil^12^), and consumption in the human diet (500 mg/day^3^). Importantly, our selected concentration exceeded the sorption limit for mineral soils (5–10 mg polyphenol/g soil^24^), ensuring bioavailability for our microbially focused studies. From the triplicate, anoxic soil reactors, 16S rRNA genes, metabolites, and genome-resolved metaproteomes were sampled on days 1, 3, 7, 10, 14, and 20 (Fig. 1c).

Our experimental design included two control treatments to (i) discern polyphenol-stimulated responses from native, background soil microbial activity and (ii) differentiate microbially-mediated CT degradation from abiotic CT degradation resulting from reactions with the soil matrix. First, to separate the impacts of polyphenols from background soil microbial processes, we performed parallel, temporal analyses on CT-amended and unamended control soil reactors (unamended control, -CT). Second, given that CT is known to abiotically react with components in soils^22^, we also amended autoclaved soil with CT (autoclaved soil, +CT). This latter control did not contain amplifiable DNA over the course of the experiment, supporting microbial-inactivation during the time course monitored in this treatment (Supplementary Note 1). While we recognize the potential for autoclaving to alter soil chemistry^25^, we show at inoculation there was little difference in the soil chemical landscape between autoclaved and unautoclaved CT-amended soil microcosms (Supplementary Fig. 2). Together these findings support the utility of autoclaved soils as a comparative metabolite control to identify microbial and soil abiotic transformations of the CT polymer. Collectively, this experimental design, analyzed with integrated high-resolution techniques, offered a new platform to resolve soil microbiota responses to polyphenols under anoxic conditions.

### Metabolomic evidence supports abiotic and biotic polyphenol degradation

Our primary goal was to monitor chemical transformations of a model polyphenol between active and inactive soil communities to discover evidence for microbiological degradation products under anoxic conditions. Prior to this research, low-resolution chemical assays (e.g. Folin–Ciocalteu for polyphenols or acid butanol for CTs) were commonly used to assess polyphenol content in soils^12^. However, the Folin-Ciocalteu method suffers from a lack of specificity because it measures oxidizable substrates, such as polyphenols, but also including a variety of organic and inorganic constituents of biological systems including soils^26,27^. Further, the acid butanol method is highly specific for CT, but responds poorly to CT in soils or other complex matrices because of interfering interactions between CT and protein or particulates^28,29^. It is also not possible to detect structural changes to the CT polymer using the acid butanol assay, as for example it does not respond differentially based on degree of polymerization^30^. Beyond analytical methods, most earlier studies amended soils with crude mixtures of polyphenols (e.g. leaf extracts^31^) with these mixtures likely obscuring identification of polyphenol degradation products, while other studies lacked microbially-inactivated controls that likely prohibited clear assignment of degradation products to microbial processes. Here, we used a structurally-defined CT polymer (Fig. 1b) and employed multiple control treatments, while using various high-resolution metabolomic techniques to track CT depolymerization and degradation products over time.

**Fig. 2:**
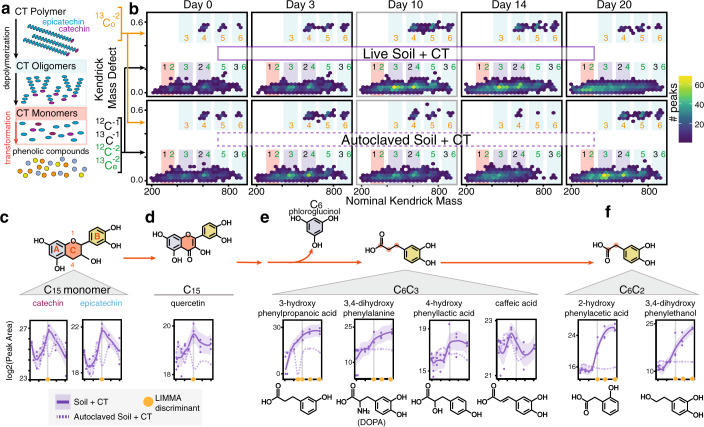
Metabolomic evidence for anoxic degradation of a model polyphenol in soil. **a** Model CT polymers have an average degree of polymerization of 16, with repeating epicatechin (blue) units capped with a terminal catechin (magenta). Depolymerization breaks the interflavan bonds of the polymer backbone, generating smaller sized oligomers and monomers. These can be further transformed, by biotic or abiotic processes, to lower molecular weight phenolic compounds. **b** Kendrick mass defect (KMD) hex plots for the peaks detected in replicate C of biologically-active (top) and autoclaved (bottom) CT-amended microcosms. KMD is given relative to (epi)catechin. Hex plots divide plot area into equal size hexagons, and hexagons are colored according to the number of data points that fall in that area. At left, peak information that enabled oligomer assignments is shown by colors where ^13^C_o_ (orange) and ^13^C_e_ (green) denote doubly-charged compounds containing odd and even numbers of ^13^C, respectively (see Supplementary Fig. 4 for detailed examples). Colored rectangles are shown around regions where CT oligomers (blue) and monomers/dimers (pink) and their derived compounds are expected to occur, with key regions highlighted in purple. Corresponding colored numbers indicate oligomer sizes: monomer (1), dimer (2), trimer (3), tetramer (4), pentamer (5), and hexamer (6) peaks. Kendrick plots for all replicates at all timepoints are found in Supplementary Fig. 6A–C. **c–f** Metabolites detected via LC–MS can be organized into **c** CT C_15_ monomers (epicatechin, catechin), **d** other C_15_ flavonoids (quercetin), **e** C_6_-C_3_ phenolic compounds, and **f** C_6_-C_2_ phenolic compounds. Metabolite dynamics are shown with lines indicating average peak area (*n* = 3 individual biological replicates) for CT (purple) microcosms, and shaded areas the 95% confidence interval with individual data points plotted. Dotted lines show signal from autoclaved CT-amended soil control. Orange circles indicate timepoints at which active soil signal significantly differed from autoclaved soil signals (LIMMA, *p* < 0.05, log2FC > 1.5, see Supplementary Data 1 for exact p-values). Vertical gray lines mark day 10. In the illustration of the monomer structure in (**c**), red letters label flavonoid rings, and red numbers correspond to C-ring position.

We first wanted to observe changes at the CT-oligomer level over time, with the temporal increase in smaller oligomers indicating depolymerization (i.e. interflavan bond cleavage, Fig. 2a) of the larger CT polymer. Our Fourier-Transform Ion Cyclotron Resonance Mass Spectrometry (FTICR-MS) analysis captured nearly 90,000 peaks across all samples that corresponded to compounds in a specific relatively high molecular weight mass range. Within these peaks, we developed a workflow that identified peaks corresponding to CT oligomers and transformation products (Supplementary Fig. 3). We carried out subsequent Kendrick Mass Defect (KMD) analysis on these CT peaks using (epi)catechin as the base unit (described in Supplementary Fig. 4). This KMD analysis resolved CT oligomers and derived compounds without assigning chemical formulas (Fig. 2b). Within a single KMD plot, the distribution of epi(catechin) oligomers and derived compounds ranging from monomers to hexamers were visualized (Fig. 2b). Clouds of points were separated along a horizontal axis by oligomer size (mass). Importantly, Kendrick plots for multiply-charged polymers separate along a vertical axis based on an “isotopic split”^32^. In this phenomenon, Kendrick plots of polymers at charge state *z* exhibit *z* clear horizontal lines separated by 1/*z* KMD^32^ (Supplementary Fig. 4). Therefore, in our Kendrick plots, the two horizontal lines separated by ~0.5 KMD indicated that we had two subpopulations of polymer oligomer species in our spectra with −1 and −2 charge. The primary horizontal line (KMD ~0) corresponds to the singly- or doubly-charged ^12^C monoisotopic species, the singly-charged ^13^C species, and the doubly-charged ^13^C_2, 4, …_ species. The separated peaks at ~0.5 KMD represent the doubly-charged ^13^C_1,3,5.._ -containing species (Supplementary Fig. 4). Synthesizing mass data and inferring −1 or −2 charge, we identified peaks on the Kendrick plot in oligomer size regions where points corresponding to oligomers and their transformation products (ex. degradation intermediates) can be found (Fig. 2b, blue, purple, and pink rectangles).

The Kendrick plots for live and autoclaved CT-amended reactors show the presence of CT oligomers and transformation products ranging in size from monomer-hexamer at all timepoints. We confirmed the CT polymer did not contain detectable CT monomers or other flavonoids (e.g. quercetin) in its pure form prior to amendment (Supplementary Fig. 5). Therefore, the appearance of monomer peaks on day zero in both live and autoclaved soil microcosms suggests either low levels of monomers were present in the soil sample, or that abiotic processes originating in the soil microcosm generated some monomers soon after adding the polymer to the soil. In looking at Kendrick plots generated for the live, unamended control soils (Supplementary Fig. 6c), we recovered negligible peaks that could be attributed to CT oligomers or monomers, thus supporting the likelihood that abiotic reactions between CT and the soil matrix, and not background soil CT concentrations, were sources for these compounds at day 0 in the CT-amended samples.

The Kendrick plots for biotic soils revealed a marked increase in the number of peaks corresponding to CT oligomers and transformation products (Supplementary Fig. 6d) at days 10 and 14. Although the autoclaved soil control reaches the same level of richness by day 20, the rate of CT transformation is enhanced in biotic microcosms. Further supporting this, in biotic soils there is an increase in smaller CT oligomers (e.g. CT tetramers, trimers, dimers, and monomers) over time and particularly at day 10, a trend not observed in the autoclaved soils until day 20 (Fig. 2b, purple rectangles). Further supporting the accumulation of smaller oligomers (< 6-mer) in microbially-active soils, the peaks detected in the 0.5 KMD region increased in the biotic relative to autoclaved soils (Fig. 2b). Peaks in this region likely derive from naturally-occurring ^13^C-containing compounds that are only detected when the parent ^12^C peaks from equivalent compounds are highly abundant^33^ (Fig. 2b, Supplementary Fig 4). This latter finding further supported that biotic microcosms contained more CT depolymerization and transformation products (from monomers to hexamers) than the autoclaved control (Fig. 2b) at later timepoints, signifying that microbiota in the soils contributed to CT depolymerization. These microbially-enabled depolymerizations were in addition to abiotic transformations of the CT polymer that were observed in the autoclaved samples within a twenty-day period.

Given FTICR-MS indicated CT depolymerization to smaller oligomers and monomers over time in microbially-active soils, we tracked the production of CT phenolic monomers (e.g., epicatechin or catechin) and subsequent degradation of these monomers using liquid chromatography–mass spectrometry (LC–MS). The LC–MS data supported the FTICR-MS data, providing additional evidence for CT depolymerization, as the monomers were detected in the biotic and autoclaved soils at all timepoints (Fig. 2c). Pairwise comparisons of the biotic and abiotic data indicated that C_15_ monomers epicatechin and catechin were significantly enriched at day 10 in the biotic incubations (Fig. 2c). In parallel, we also detected the C_15_ flavonoid quercetin^34^ in both treatments, but like the CT monomers it was also only significantly enriched in microbially active reactors at day 10, supporting the biotic production of this compound from (epi)catechin as others have postulated^34^. Thus, consistent with our FTICR-MS findings, we see an enrichment of C_15_ monomers and close derivatives occurring in the microbially-active soils midway through the experiment (day 10). Together, our FTICR and LC mass spectrometry approaches contributed to a model where the interflavan bonds in the CT polymer were broken from a contribution of biotic and abiotic processes, yielding shorter CT oligomers and CT monomers catechin and epicatechin. These data contradict the long-standing dogma in soils that the interflavan bonds linking monomers in the CT polymer are stable under anoxic conditions^5,7,35^.

Importantly, the C_15_ flavonoids (epicatechin, catechin, or quercetin) decreased in abundance after day 10 only in microbially-active soils, suggestive of further biodegradation (Fig. 2e). Based on our metabolite identifications in the CT-amended live and autoclaved soils, it is likely these flavonoids underwent heterocyclic C-ring fission (position 1 and 4 orange ring, Fig. 2c, d) to generate a C_6_ compound (phloroglucinol, blue ring) from the A-ring and C_6_–C_3_ acid from the B-ring and C-ring carbon atoms (Fig. 2e). The C_6_–C_3_ acid can be envisioned as the parent (e.g., by loss of CO_2_) of phenylacetate derivatives (C_6_–C_2_, Fig. 2f) and several putatively identified benzoic acids (C_6_–C_1_) and simple phenols (Supplementary Data 1)^36^.

With decreased abundance of C_15_ flavonoids from microbial degradation, we observed a concomitant increase in many downstream phenolic metabolites in the microbially-active CT treated soils. Specifically, (i) four phenolic metabolites (C_6_–C_3_ and C_6_–C_2_) were significantly enriched at multiple timepoints (Fig. 2e, f), (ii) two C_6_–C_1_ and C_6_ metabolites were significantly enriched at day 20 (possibly 4-methylcatechol, hydroquinone; Supplementary Data 1), and (iii) another 3 phenolic metabolites were uniquely detected via NMR (C_6_–C_3_ phenylpropionic acid, C_6_–C_2_ 3,4-dihydroxyphenylacetic acid, C_6_–C_2_ 3-hydroxyphenylacetic acid; Supplementary Data 1). The flavonoids and phenolic compounds identified by LC–MS and NMR had differing dynamics between our biotic and abiotic controls, indicative of unique production from microbial activity, and they were present in relatively negligible amounts in the unamended controls (Supplementary Fig. 6), further indicating that these products derived from the added CT. Therefore, accounting for differences between the biotic samples and both control reactors, we concluded that the smaller phenolic compounds derived from microbial biodegradation of the added CT (Fig. 2a).

Detection of some phenolic compounds could not be statistically resolved between microbially-active and autoclaved treatments, although they were generally less abundant in the autoclaved controls (Fig. 2e). This result pointed to abiotic processes as additional transformers of CT monomers in anoxic soils. As the C_15_ monomer was transformed, the LC–MS data suggested the 3,4-dihydroxylation pattern of the parent flavonoid compound B-ring was retained across biotic and autoclaved soils (Fig. 2e, f). However, in microbially-active microcosms we also detected compounds with altered hydroxylation patterns, suggestive of distinctly biotic transformations: dehydroxylation yielding 3-hydroxy derivatives (Fig. 2e), or rearrangement to yield a 2-hydroxy derivative^37^ (Fig. 2f). Also in these microbially-active soils, we detected a phenolic amine, 3,4-dihydroxyphenylalanine (DOPA), that was enriched significantly at later timepoints (phase 3) (Fig. 2e). Collectively, this variety of phenolic metabolites detected in the later phases reinforced our hypothesis that while abiotic transformations of CT occurred in our anoxic soils, there were clear signals of microbial CT and monomer biodegradation that occurred on different time scales and yielded unique products.

Broadly, the fate of the CT polymer in microbially-active anoxic soils paralleled some polyphenolic transformations reported in mammalian fecal metabolomes^38^. We observed increased caffeic acid (Fig. 2e) and putative dihydroxybenzoic acids (e.g., vanillic acid) (Supplementary Data 1), which are suggested metabolite biomarkers^39^ for anoxic polyphenol degradation in feces. Yet these proposed biomarkers were also detected in our autoclaved CT-amended soils, further reinforcing the need to partition abiotic and biotic processes when working in chemically complex matrices like feces or soil. A more detailed analysis of the shared and unique features of polyphenol degradation in soils compared to the human gut is provided (Supplementary Fig. 7, Supplementary Note 2).

In summary, we provided chemical evidence for polyphenol degradation in soils under anoxic conditions. With support from multiple analytical methods, we concluded that CT likely underwent abiotic transformations, yet distinct increases in CT oligomers, monomers, and putative biodegradation products over time were detected only in microbially-active soils. This highly-resolved metabolite data provides a chemical framework for microbial polyphenol degradation in anoxic soils, a scaffolding that can be leveraged in future, more targeted, research using varied polyphenol substrates, as well as across a wider range of soil types and conditions.

### Genome-resolved metaproteomics reveals enrichment of polyphenol responsive microbes

**Fig. 3:**
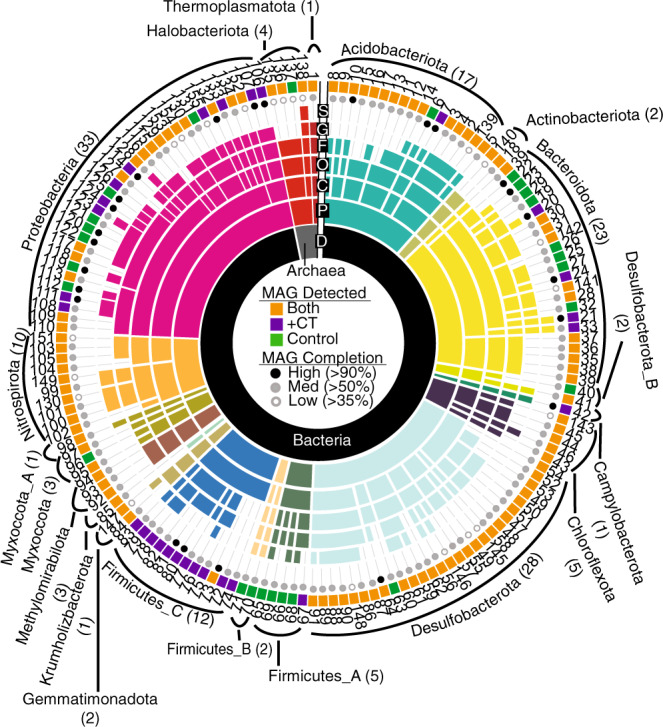
Taxonomy and detection of the 155 dereplicated metagenome-assembled genomes (MAGs) in the soil reactor genome database. Sequential colored rings indicate the most resolved taxonomic level that could be assigned by GTDB-tk. Taxonomic level (Domain, Phylum, Class, Order, Family, Genus, Species), is denoted in black with a single letter abbreviation. Ring color corresponded to phylum assignment, with the phylum listed on the outside with the number of dereplicated MAGs in parentheses. Circles at the sunburst edge summarize genome completion, while the listed number is the MAG ID (see Supplementary Data 2). Colored rectangles at the sunburst edge indicate MAG distribution across treatments, with MAGs detected (see Methods for thresholds) only in CT (purple), or only in control (green), or from both conditions (orange) denoted.

In light of our metabolite data indicating active microbial polyphenol degradation, we next explored the impact of polyphenols on the soil microbiome. To uncover the key microbial players and functions underlying anoxic polyphenol responses in our soil reactors, we constructed a genome database composed of metagenome-assembled genomes (MAGs) from CT-amended and unamended samples at various timepoints. Specifically, metagenomic sequencing from the microcosms at days five, ten, and twenty were obtained (Fig. 1c), totaling 500 Gbps sequencing (Supplementary Data 2). This sequencing depth represents 9-fold more sequencing per sample compared to published field wetland metagenome studies to date, thereby increasing the sensitivity for detecting the breadth of microbial functions encoded in these soils^40^ (Supplementary Fig. 8). From this sequencing, we assembled and reconstructed 294 MAGs, which were dereplicated at 99% average nucleotide identity into 155 MAGs (Fig. 3, Supplementary Data 2), of which 87% were medium- and high-quality genomes^41^ (Fig. 3, Supplementary Data 2). Based on read mapping to this soil-derived MAG database, the majority (65%, *n* = 101) of genomes were present across treatments. Despite the extensive depth of sequencing, 17% of genomes (*n* = 26) were only recovered in non-CT reactors, while 18% (*n* = 28) of genomes were only recovered from CT reactors (Supplementary Fig. 9). Importantly, of these CT-amendment specific MAGs, just 29% (*n* = 8) were recovered at every sampled timepoint, highlighting the need for time-resolved metagenomes to capture community MAG composition in soil microcosms. The dereplicated MAG database evenly recruited metagenome reads across the samples, indicating there was little bias in assembly and binning due to treatment type (Supplementary Fig. 10). This dataset illustrated the value of targeted amendments, temporal sampling, and deep sequencing for bringing to light conditionally rare taxa that may have ecosystem-relevant metabolic capabilities^42^.

The dereplicated MAG database (*n* = 155) contained genomes from 19 phyla, many of which represent the most abundant and cosmopolitan lineages in soils^40^ (Fig. 3). However, using the Genome Taxonomy Database toolkit (GTDB-tk)^43^, we found that a subset of our genomes represented newly sampled lineages (5 orders across 3 phyla), and a large proportion of our MAGs belonged to lineages defined only by alphanumeric identifiers in the GTDB at the class (17%), order (6%), or family (21%) levels (Supplementary Data 2). Further stressing the phylogenetic novelty in these soils, less than 1% of our soil microcosm 16S rRNA amplicon sequencing variants (ASVs) had similarity (>97%) to 16S rRNA genes represented in RefSoil^44^ (a database of soil isolate genomes) (Supplementary Table 1). The discrepancy between genomes uncovered in these soil microcosms and those included in public soil genome databases underscores the need for establishing study- or site-specific genome databases for uncovering cryptic biochemistry in soils.

**Fig. 4:**
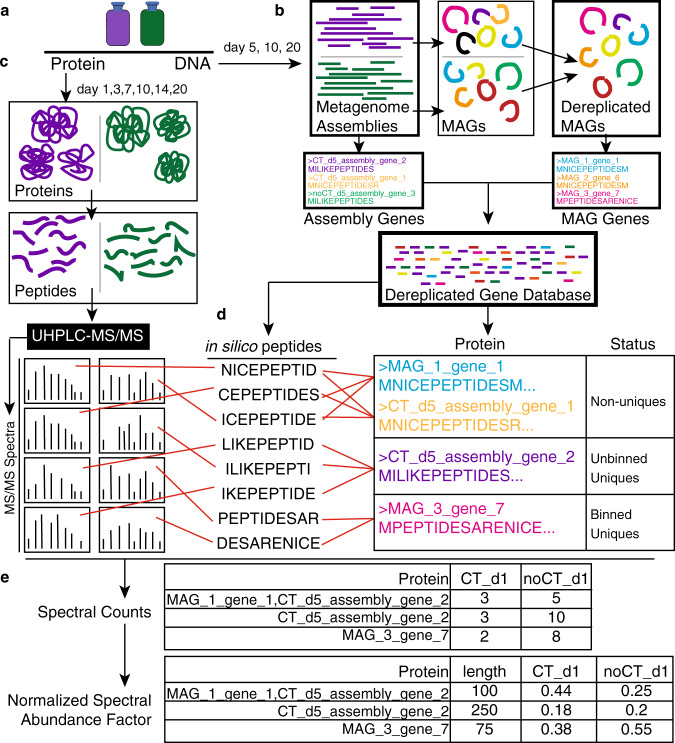
Workflow for genome-resolved metaproteomics. **a** DNA and proteins were sampled from triplicate live soil + CT (purple) and unamended soil (green) reactors at days 5, 10, 20 (DNA) and days 1, 3, 7, 10, 14, 20 (protein). **b** Metagenomes at each timepoint were obtained for both CT (purple) and unamended (green) treatments. Metagenomes were assembled and binned to obtain metagenome-assembled genomes (MAGs) across all samples. This set of MAGs was dereplicated at 99% ANI to obtain a MAG database of 155 dereplicated MAGs (Fig. 3). Using amino acid translations of genes derived from this dereplicated MAG database and remaining genes from metagenomic assemblies (on unbinned scaffolds >2500 bp), we compiled a Dereplicated Gene Database (all unique gene sequences) that served as our reference database for our metaproteomes. **c** Metaproteomes at each timepoint were obtained as described in Methods. **d** Spectral matching was carried out using obtained spectra and in silico spectra derived from the gene database. From this, proteins were classified as “non-unique” if the recruited peptides could be derived from other proteins in the database. Proteins were classified as “unbinned uniques” if they had peptides that could only be matched to the amino acid sequence derived from a metagenomic unbinned scaffold in our assembly. Proteins were identified as “binned uniques” if they had peptides that could only be matched to that amino acid sequence, and were derived from a single genome in our MAG database. **e** All identified proteins were quantified with label-free spectral counts. This was then corrected for protein length and sample-to-sample variation by conversion to normalized spectral abundance factor.

**Fig. 5:**
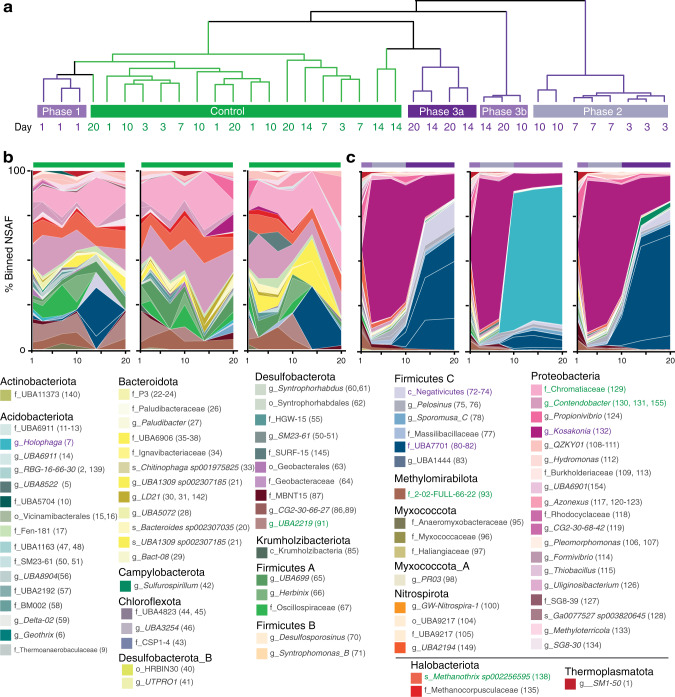
Polyphenol-amended soil microbial communities showed distinct and dynamic metaproteome responses. **a** Hierarchical clustering of MAG-contributions (binned uniques) to metaproteome samples. Unamended control metaproteomes are shown in green, while the multi-phase response of CT-amended microcosms are highlighted with varying shades of purple. **b**, **c** Genome-resolved metaproteomic dynamics in CT-amended microcosms. The relative contribution of MAGs to the binned unique peptide pool is shown for the three unamended **b** control and **c** CT microcosm replicates over 20-days. The most refined GTDB-tk assigned taxonomy is listed by phylum, with our MAG ID number in parentheses (Supplementary Data 2). The names of the top 5 peptide-recruiting MAGs are colored for CT (purple) and control (green) microcosms. Supplementary Fig. 11 shows this data with the total metaproteome data, including unbinned uniques, binned uniques, and non-uniques.

To maximize the recovery of functions in our metaproteome analysis, we combined genes from all metagenomic assemblies, including binned genes from our MAG database and unbinned genes from metagenomic assemblies (Fig. 4c), to build a representative dereplicated (100% amino acid identity) gene database. Importantly, we verified changes in observed peptide recruitment derived from changes at the peptide level rather than a database effect (Supplementary Fig. 10, Supplementary Note 3). After mapping the metaproteomes obtained from CT-amended and unamended microcosms at six timepoints to our dereplicated gene database (*n* = 36), we recovered 11,942 peptides that mapped to 50,446 potential proteins (Supplementary Data 3). From here, proteins were categorized into three groups based on if the peptides were unique to specific genomes (Fig. 4e, categories detailed in Methods). Nearly 60% of the recovered peptides were uniquely recruited to 119 of 155 dereplicated MAGs (known as “binned uniques”, see Methods), enabling identification of active community members in our genome database over time (Fig. 5, Supplementary Fig. 11). Notably, 47 MAGs recruited peptides exclusively in CT-microcosms, while just 3 MAGs were inferred to be active exclusively in unamended control soils. Alternatively, the remaining 69 MAGs recruited peptides in both CT and unamended microcosms, hinting at the metabolic plasticity harbored in soils.

Mirroring trends in microbial 16S rRNA gene composition and exometabolite changes over time (Supplementary Fig. 12), metaproteomes of CT and unamended control microcosms diverged temporally (Fig. 5a, Supplementary Data 3). The gene expression of members in the unamended control were relatively stable across the experimental period (i.e. no temporal clustering, Fig. 5a, b). MAGs belonging to members of Chromatiaceae, *Contendobacter*, *Methanothrix*, MBNT15, and Methylomirabilota recruited 50% of binned unique peptides in the unamended control reactors. Collectively these MAGs accounted for less than 5% of binned unique peptides in the CT treatment, indicating the capacity for the polyphenol to shift active populations in soils under our study conditions. While this represents one of the first reports on the impacts of polyphenols on soil microbial community gene expression, similar temporal shifts in microbial community 16S rRNA gene membership have been observed with complex and pure polyphenols in soils and guts^15,45,46^.

In contrast to the unamended controls, CT-amended soils displayed a multi-phase gene expression response (Fig. 5a–c). In phase 1, metaproteomes from CT treatments at day 1 could not be differentiated from unamended controls (Fig. 5a). In phase 2 (days 3–10), a MAG from the Proteobacterial genus *Kosakonia* (CTSoil_132, dark purple) accounted for 80% of the binned unique peptides from the CT-treated samples, with peak gene expression observed on day 3 (Fig. 5a, c). In phase 3 (day 10–20), while *Kosakonia* expression was still detected, the CT-amended reactor metaproteome replicates displayed heterogenous responses (Fig. 5a, c, Phases 3a and 3b), dominated by either a novel member of the Acidobacterial genus *Holophaga* (CTSoil_7, teal) or three novel MAGs in the Sporomusales undescribed family UBA7701 (CTSoil_80, CTSoil_81, & CTSoil_82, dark blue). By genome-wide average amino acid identity and ribosomal protein similarity, these three Sporomusales MAGs likely represent three different genera (Supplementary Fig. 13). Of these three MAGs, CTSoil_81 was dominant across the metaproteome data, recruiting four-times more peptides than the other two Sporomusales. While we did detect peptides from these three dominant MAGs (*Kosakonia*, *Holophaga*, or Sporomusales CTSoil_81) in non-CT amended controls, these were annotated as primarily housekeeping (e.g. RNA polymerase) or hypothetical proteins (Fig. 5b). Together, this suggested that while these microorganisms may have subsisted from metabolisms independent of polyphenols, they demonstrated different functionality under polyphenol exposure. Based on these findings, we concluded members of these 3 taxa were stimulated by polyphenols in anoxic soils. As such, we sought to link the metaproteome functions of these taxa and the broader microbial communities to our polyphenol degradation metabolite scheme.

### Polyphenol biodegradation occurs through metabolic exchange in anoxic soil

**Fig. 6:**
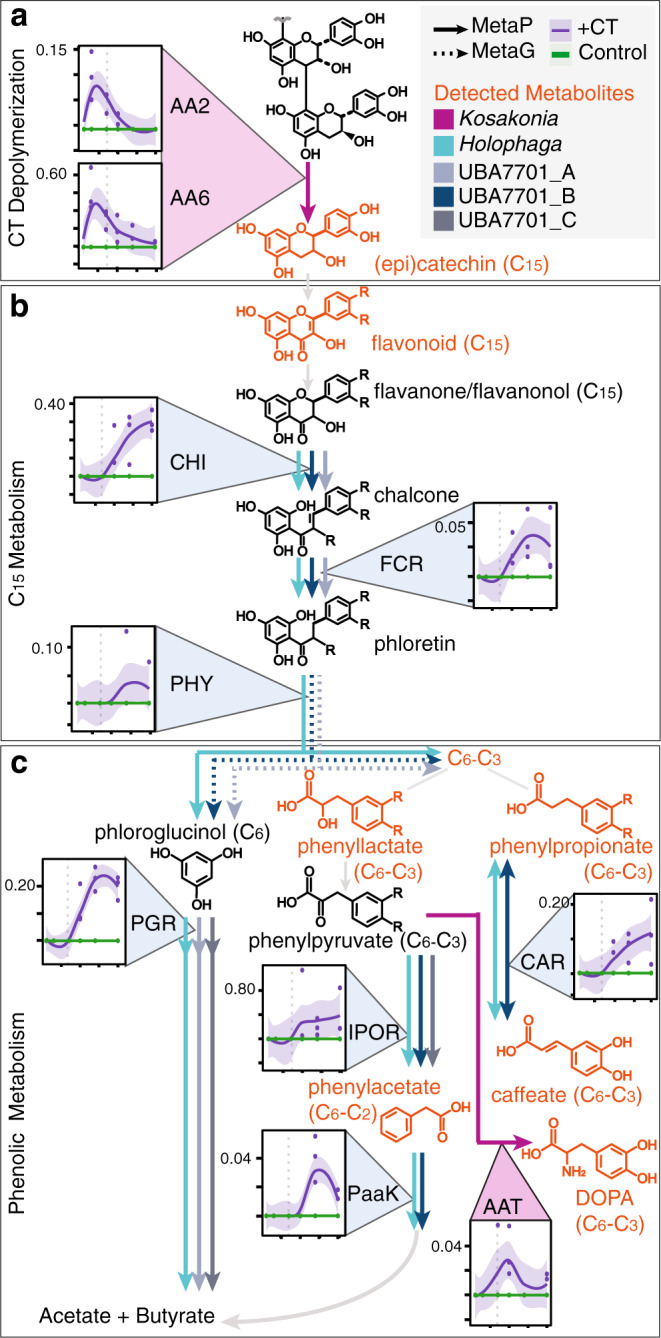
Metaproteome data supported polyphenol degradation by *Kosakonia, Holophaga*, and Sporomusales UBA7701. Line graphs in **a**–**c** indicate average % normalized spectral abundance factor (NSAF) with shaded areas denoting the 95% confidence intervals for CT (purple, *n* = 3 individual biological replicates) and unamended (green, *n* = 3 individual biological replicates) soil microcosms, with individual data points plotted. Dotted vertical lines are shown to mark day 7 across plots, demarcating phase 1 and 2 from phase 3. Phenolic compounds in orange are detected in metabolomics, with arrow color corresponding to MAGs expressing detected enzymes. Dotted arrows represent metagenome-encoded enzymes. **a** CT-depolymerization may be mediated by peroxidase activity from AA2 and indirect activity from AA6 expression from *Kosakonia*, **b** C_15_ biodegradation may be performed by the coordinated activity of three enzymes (CHI, FCR, PHY), these gene sets are expressed both by *Holophaga* and Sporomusales UBA7701 MAGs, and **c** multiple phenolic-active enzyme dynamics expressed by MAGs from these two taxa likely yield energy and produce acetate and butyrate. Enzyme abbreviations are as follows: peroxidase (AA2, EC 1.11.1.21); 1,4-benzoquinone reductase (AA6, EC 1.6.5.6), chalcone isomerase (CHI, EC 5.5.1.6), flavanonol-cleaving reductase (FCR), phloretin hydrolase (PHY, EC 3.7.1.4), phloroglucinol reductase (PGR, EC 1.3.1.57), caffeoyl-CoA reductase (CAR, EC 1.3.1.108), indole-pyruvate oxidoreductase (IPOR, EC 1.2.7.8), phenylacetate-CoA ligase (PaaK, EC 6.2.1.30), and aromatic amino acid aminotransferase (AAT, EC 2.6.1.57).

Metabolite evidence indicated soil microbiota depolymerized CT in the first 10-days (Fig. 2), consistent with when *Kosakonia* was most active via metaproteomics (Fig. 5). Given the size of the CT polymer, we expected any microbial depolymerization to be extracellular and thus we were particularly interested in the expression of two putatively-secreted enzymes from *Kosakonia* during this phase. One of these enzymes, a peroxidase (AA2) has been biochemically demonstrated to aerobically degrade phenolic-rich lignin polymers^47^, while the other, a 1–4,benzoquinone reductase (AA6), is known to be indirectly involved in lignin degradation^47^ (Fig. 6a). The peroxidase, a predicted katG*-*type, uses H_2_O_2_-derived radicals to carry out 1-electron oxidations of a chemical mediator—potentially a phenolic compound or Mn^2+^ ^48^. This extracellular low molecular weight mediator can diffuse to react with substrates outside the enzyme’s spatial range. The benzoquinone reductase can participate in Fenton cycling to support H_2_O_2_ pools^47^ (Fig. 6a). Analogous to what is proposed for aerobic lignin degradation, these oxidations could generate radical sites within the CT that promote depolymerization through cascades of bond scissions^47^. Further supporting this proposed role in anoxic CT depolymerization, these two enzymes (AA2, AA6) were recently implicated in pure-culture, anaerobic lignin degrading experiments by a close relative of *Kosakonia*^49,50^. As lignin is also a complex polyphenolic polymer, it is reasonable to extend the roles for these lignin associated enzymes to include CT depolymerization. Given these tantalizing shared findings at both the soil microcosm and isolate levels, biochemical characterization of these enzymes is warranted to expand roles for these canonically aerobic enzymes into anaerobic polyphenol degradation.

Analysis of the *Kosakonia* genome failed to detect known phenolic compound biodegradation pathways, suggesting *Kosakonia* enrichment is not fueled by phenolic catabolism. In support of this, during phase 1 and phase 2 we detected simultaneous expression of genes for sugar transport (e.g. maltose, fructose sugar phosphotransferase systems), central carbon metabolism, and acetate production (Supplementary Fig 14). Thus, it is possible that *Kosakonia* performed CT transformation for chemical detoxification, not energy-generation, while co-metabolizing sugars fermentatively^51^. In support of this *Kosakonia-*mediated detoxification, expression of genes for two previously observed mechanisms of CT tolerance were detected: RND-type transporters, to remove toxic phenolics from the cell^52^, and Spy proteins, thought to maintain cell membrane integrity in response to CT-induced environmental stress^53^. Collectively, this time-series expression data paired to high-resolution metabolite products during phases 1 and 2 (days 1–10) signified *Kosakonia* detoxified CT while fermenting sugars, ultimately serving as the most likely candidate for CT depolymerization in the live soil microcosms.

*Kosakonia* is also the most likely candidate for DOPA production, a C_6_-C_3_ phenolic amine metabolite that was significantly produced in the microbially-active soils at later timepoints (Figs. 2e and 6c, purple arrow). We suggest *Kosakonia* produced DOPA via an aromatic amino acid aminotransferase, that was exclusively produced by *Kosakonia* in phases 2 and 3 when DOPA was produced (Supplementary Fig. 15). Plant root exudation and litter decay are commonly considered the primary source of soil DOPA, where this compound has broadly antagonistic allelochemical properties^54^. As an alternative source of DOPA in soils, our plant-free microcosms highlight that microbes could produce this compound from polyphenol-derived phenolics (Fig. 2e). Beyond soils, this result may have cross-ecosystem ramifications. If similar microbial biochemistry occurs in the gut, DOPA could be microbially produced from dietary polyphenols, which could cross the blood-brain barrier and be converted to dopamine by host enzymes^55,56^, providing a plausible rationale for the positive gut-brain connection with polyphenol-rich foods (e.g. wine, chocolate)^57^.

Next, we investigated metabolic roles of microorganisms in the latter half of our experiment that could support the proposed biodegradation scheme where C_15_ flavonoids (epicatechin, catechin, quercetin) were converted to smaller phenolic acids (Fig. 2c–f). During this time, metaproteomic data implicated increased activity of a MAG affiliated with *Holophaga* and three MAGs (CTSoil_80–82) within the Sporomusales family UBA7701 (Fig. 5c). In comparing to known flavonoid degrading enzymes, proteome profiles from *Holophaga* and the Sporomusales UBA7701 MAGs showed these MAGs likely carried out the transformations observed in our metabolite data.

The first enzyme in this proposed flavonoid monomer degradation pathway was a chalcone isomerase (CHI), which could generate a chalcone^58^ from opening the C-ring (position 1) of quercetin, a C_15_ flavonoid detected only in our microbially active soils, likely from (epi)catechin monomers (Fig. 6b). This chalcone could be reduced to phloretin by a second enzyme, a NADH-dependent flavanone- and flavanonol-cleaving reductase^36^ (FCR, Fig. 6b). C-ring cleavage is then completed with release of C_6_ phloroglucinol and C_6_-C_3_ acids by a third enzyme, phloretin hydrolase^59,60^ (PHY, Fig. 6b). While we recovered CHI and FCR from both *Holophaga* and two Sporomusales MAGs (CTSoil_80 & 81), peptides for the last enzyme PHY were only confidently detected from *Holophaga* (Fig. 6b), however the two Sporomusales MAGs encode this gene in their genome (CTSoil_80 & 81, Fig. 6b dotted line). This microbially produced suite of enzymes likely catalyzed the degradation of the CT-oligomer derived flavonoids to other phenolic compounds observed after day 10 (Fig. 2a).

While we note these enzymes (CHI, FCR, PHY) were first uncovered and described in flavonoid-degrading gut microbial isolates^6^, they remain poorly annotated in KEGG (and other databases) remaining as “hypothetical”, or non-specific classes like “oxidoreductases”. As such, we used non-homology-based annotation approaches, including coordinated gene expression-metabolite data combined with structural protein modeling, to inform these gene annotations (Supplementary Fig. 16, Supplementary Data 3). To the best of our knowledge, this is the first report of these enzymes in soil-derived microorganisms, collectively illustrating the ways that currently cryptic processes in soil can be informed by cross-ecosystem analyses from more tractable microbiomes.

Together these multi-omics data provided evidence for the biodegradation of CT monomers and their derivatives to phenolic acids (specifically phloroglucinol and C_6_-C_3_ acids) by *Holophaga* and members of the Sporomusales. We note, the C_6_ metabolite phloroglucinol was not detected in our exometabolites, but this was consistent with its typical rapid entry into primary metabolism^5^. Moreover, *Holophaga* and Sporomusales MAGs expressed putative phloroglucinol reductases (PGR), the key enzyme for phloroglucinol degradation via an energy-generating pathway producing acetate and butyrate^61^ (Fig. 6c).

In addition to monomer (C_15_) degradation, both *Holophaga* and the Sporomusales UBA7701 expressed several enzymes that carry out other phenolic transformations (Fig. 6c). For example, from days 10–20, both *Holophaga* and UBA7701 MAGs expressed indole-pyruvate oxidoreductase, which could reduce C_6_-C_3_ phenylpyruvates to observed C_6_-C_2_ phenylacetates^62^ (IPOR, Fig. 6c). Further, they also produced phenylacetate CoA-ligase (PaaK, Fig. 6c), the key enzyme for degrading phenylacetate via an anaerobic pathway that feeds to central metabolism^63^. Lastly, and in support of a specialized form of anaerobic respiration, both *Holophaga* and Sporomusales MAGs expressed genes for the Car-system which could allow caffeic acid^64^ reduction (a C_6_-C_3_ phenolic metabolite detected in our CT reactors, Fig. 2f). Consistent with prior reports and supported by the metaproteome data, we propose these taxa couple sugar and phenolic oxidation (and maybe CO_2_ fixation, Supplementary Fig. 14), to the reduction of the abiotically-generated CT metabolite caffeate as an electron acceptor, generating 3,4-dihydroxypropionate (Fig. 6b).

Our metaproteome results illustrated the vast levels of functional redundancy that reside in soils, where members of two different phyla (Acidobacteria and Firmicutes) expressed nearly identical metabolic pathways for C_15_ flavonoid biodegradation and phenolic metabolism. Taken together, these late phase dominant members (*Kosakonia*, *Holophaga*, *Sporomusales*) expressed enzymes to metabolize a range of CT oligomers and their derived metabolites, demonstrating that this model polyphenol was accessible to soil microbiota under anoxic conditions. Ultimately, these findings illustrate the latent metabolic versatility awaiting discovery within microbiomes across soils.

### Anoxic soil carbon cycling is resistant to polyphenol amendment

Our metabolite and metaproteome data illustrated that members of the soil microbiome can degrade polyphenols under anoxia. Beyond supposed limited polyphenol degradation, the enzyme latch paradigm suggests that polyphenols suppress microbial activity under anoxic conditions by binding extracellular hydrolase enzymes (e.g. CAZymes, peptidases) and substrates (e.g., polysaccharides, proteins)^10^. Our metaproteome data indicated diverse microbial taxa were active under CT-amendment (Fig. 5), and we next wanted to explore the impacts of CT on general microbial metabolic activities.

**Fig. 7:**
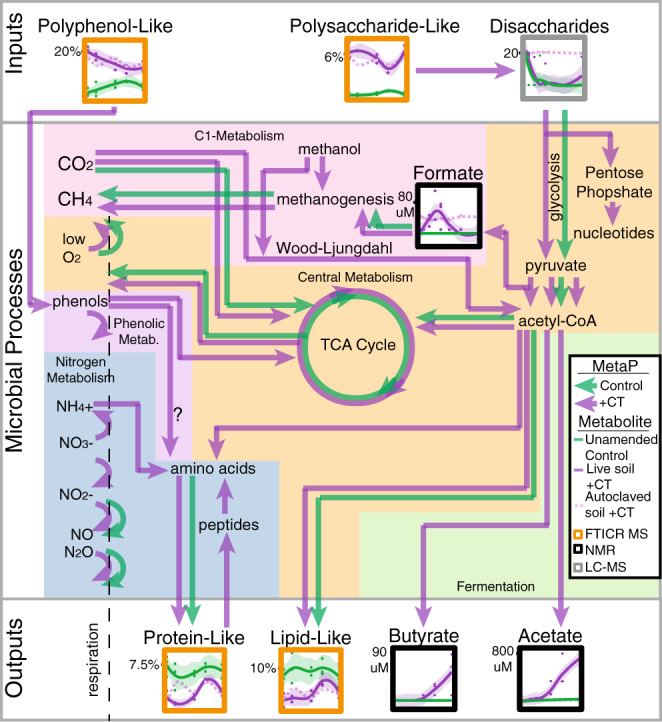
Coupled metaproteome and metabolite data indicated polyphenol amendment did not inhibit soil microbial metabolism. Arrows indicate metaproteome gene expression data, with green representing unamended and purple indicating CT-amended pathways. Metabolite dynamics are shown in boxed graphs, with lines indicating average signal for live CT (*n* = 3 individual biological replicates, solid purple), and unamended control (*n* = 3 individual biological replicates, green) microcosms with shaded areas including the 95% confidence interval and individual data points plotted, and autoclaved CT soil (dotted light purple). The methodology used to detect the metabolite is highlighted by box color (noted in the graphical legend), with FTICR-MS (orange) data given as percent of identified peaks, NMR (black) as umol, and LC–MS (gray) as log2(peak areas). Nitrogen metabolism (blue box) is discussed in Supplementary Note 4.

Additional analyses of the FTICR-MS data revealed polysaccharide-like compounds decreased over time in microbially active CT reactors (Fig. 7), findings that would not be expected if microbial activity was halted as expected by the polyphenol enzyme latch. However, we note polysaccharide-like compounds were higher initially in CT-amended microcosms and thus perhaps more available. Reasons for this could include CT amendment priming the liberation of sugars in soils^65^, or low level contamination of CT, yet the latter is not supported by an in depth molecular characterization of the pure CT^21^ (Supplementary Fig. 5). Regardless of the origin, our metaproteomic data supported increased degradation of polysaccharide-like compounds observed in the CT-amendment. We detected expression of 15 different carbohydrate-active enzymes (CAZymes) in CT reactors over phases 2 and 3, yet we did not recover peptides for CAZymes in the unamended control soils (Supplementary Fig. 14). Furthermore, we observed a corresponding decrease in LC–MS identified disaccharides over time in microbially-active CT reactors that was identical to unamended controls (Fig. 7), suggesting active carbon substrate utilization was unimpeded by CT-amendment under these anoxic conditions. Taken together, our enzyme and metabolite data did not support the enzyme latch model where polysaccharides are inaccessible to the anaerobic soil microbial community in the presence of polyphenols (Fig. 1a)^10^.

Consistent with unhindered anaerobic carbohydrate metabolism under polyphenol exposure, we observed CT-exclusive expression of sugar phosphotransferase systems (PTS, proteins used for transporting sugars into the cell), and unchanged expression of glycolytic enzymes at all timepoints (Supplementary Fig. 14). The most striking difference between CT and unamended control metaproteomes was expression of microbial fermentation pathways only under CT treatment, particularly during phase 3 when CT has been depolymerized to fermentable phenolics^66^ (Fig. 7). The CT responsive MAGs (*Kosakonia, Holophaga*, and Sporomusales_UBA7701) were inferred to be the biggest contributors to fermentative enzymes, accounting for half of the unique peptides assigned, but other members of the Firmicutes and Acidobacteria phyla also expressed these pathways (Supplementary Fig. 14). This metaproteome data was reinforced at the metabolite level, where the CT-exclusive production of formate, butyrate, and acetate was observed over time (Fig. 7). In summary, our genome-resolved metaproteomics enabled a new view of anaerobic soil microbial carbon catabolism, where polyphenol amendment did not restrict basal microbiome function.

Based on a handful of studies in the rumen, it was historically assumed that methanogens were directly inhibited by polyphenols^67^, yet recent studies have suggested the opposite may be true, as methanogen 16S rRNA genes were enriched in rice paddy field soils amended with lignin-derived phenols^68^. Here, we demonstrated that methanogenic gene expression was not impacted by CT treatment relative to unamended controls in our anoxic reactors (Supplementary Fig. 14). Methane was below 12.5 ppm in all samples (CT-amended and unamended) after 20-days. However, there were metaproteomic hits for the key methanogenesis gene *mcrA* maintained across treatments, from the acetoclastic *Methanothrix* that was implicated as the dominant methanogen in these soils under field conditions^18^, and another from a Thermoplasmatota methanogen, which was described as a key contributor to methylotrophic (C_1_-methyl) methanogenesis in these soils^20^ (Supplementary Fig. 14). As mentioned above, the methanogenic substrate acetate increased in CT-amended reactors over time, while methanol was detected in biotic and autoclaved CT-amended reactors across time, but not in unamended controls (Supplementary Data 1). Taken together, our multi-omic data failed to provide evidence that CT was toxic to these soil methanogens, and instead uncovered how abiotic and biotic CT transformations may contribute to cross-feeding these climatically relevant microorganisms in anoxic soils.

## Discussion

This study provided evidence that the anoxic soil microbiome is capable of polyphenol metabolism that includes depolymerization of a condensed tannin polymer and subsequent monomer degradation. We offered a new multi-omics enabled view of the soil microbiome’s response to a high molecular weight polyphenol under anoxia. Together our data support a model in which polyphenols in soils are not as microbially inert as previously claimed.

Importantly, our findings provide a new scaffolding that others can leverage. We expanded the definition of soil polyphenol degrading enzymes from solely (poly)phenol oxidase^9,10^, to include at least nine other enzymes (Fig. 6). Additionally, we highlighted canonically aerobic enzymes (i.e., peroxidase) that may play unrecognized roles in anoxic transformations of polymeric carbon, as has been recently suggested for other historically regarded aerobic enzymes under anoxia^49,50,69^. Our metaproteomic data unveiled the metabolic handoffs and redundancies between three anaerobic, polyphenol-responsive taxa in the soil microbial community (*Kosakonia, Holophaga, and* Sporomusales UBA7701). Finally, we showed that the underlying capacity for anaerobic carbon cycling by the soil microbiome was largely unchanged by polyphenol amendment.

We acknowledge our approach used laboratory soil reactors separated from environmental factors like fluctuating temperature, continual organic matter inputs, and interactions with micro- and macrofauna. Thus, more detailed and field-oriented studies are needed to uncover the occurrence and consequences of anoxic polyphenolic degradation under native conditions, across a range of soil types, and with different polyphenol substrates. However, here we provide initial metabolite and enzyme signatures for this process that can now be explored in greater detail in future studies.

Our study contributes to a growing body of recent research dispelling long-held notions of soil microbiomes as being intractable due to their chemical and biological hetrogeneity^17,70^. By employing multiple metabolite approaches, we tracked the transformations of a defined polyphenol along a molecular weight gradient and biochemical hierarchy: from FTICR-MS-identified oligomers, to LC–MS-identified flavonoids and phenolic acids, to NMR-identified fermentation products. Moreover, our metabolite findings echoed one another across methods (i.e., mutual detection of monomers at day 10 between FTICR-MS and LC–MS), allowing for seamless tracking of metabolites in soils. On top of this resolved view of carbon chemistry, we overlaid microbial community-wide proteome data, linking transformations of structurally-defined metabolites to enzymes that were uniquely assigned to specific genomes. We highlight the potential for the tools used here, along with a suite of other emerging technologies^71–74^, to illuminate soil microbiological and chemical processes historically confined to the “black box” of soil biochemistry.

Beyond the boundaries of these laboratory reactors, polyphenols have long been thought to act as controllers of global soil carbon storage^75^. In fact, several recent studies have suggested polyphenol-supplementation as a strategy to prevent carbon loss in peatlands^7,10,11^. However, in light of the genome-resolved metaproteome and metabolite evidence from this study, the extent that polyphenols sequester soil carbon warrants further investigation. While our study demonstrated that under anoxic conditions the soil microbiome in a freshwater wetland can degrade polyphenols and bypass proposed polyphenol locks on carbon cycling, translating this finding to climate mitigation strategies, especially relevant to peat systems, requires: quantifying the kinetics and environmental constraints of these transformations on the overall carbon budget, expanding research to other relevant polyphenol substrates, and investigating the effects of abiotic and biotic polyphenol transformations associated with diverse soil types. Our findings pave a way for these research avenues, providing metabolite and enzyme framework for mining these processes from complex systems. Collectively, our results highlight the promise of modern soil microbiome technologies for uncovering the ecological and biochemical mechanisms underlying long-held soil biogeochemical paradigms.

## Methods

### Soil sample collection

We used a soil sample collected from a plant-covered mudflat (August 2015) in Old Woman Creek National Estuarine Research Reserve^18^ (OWC) (41°22′N 82°30′W). The soil sample was stored at −20 °C until use. While we recognize that thawing these frozen soils for use in the laboratory may have impacted soil carbon availability, these soils routinely experience freeze thaw throughout the winter months and thus are exposed to fluctuating temperatures.

### Condensed tannin purification

The broad class of plant secondary metabolites known as polyphenols includes three types of high molecular weight compounds, the lignins, the hydrolysable tannins and the condensed tannins^76^. Lignins are highly methoxylated derivatives of the C_6_-C_3_ phenylpropanoids, and their fate and effects in soils have been extensively examined^47^. The unmodified phenolic moieties of tannins make these compounds more highly reactive than lignin, including their ability to serve as antioxidants, as metal binding agents, and their quintessential property of protein binding/precipitation^77^. Of the two classes of tannins, the hydrolysable tannins are highly susceptible to chemical and enzymatic decomposition via hydrolysis of ester linkages, and their metabolic fate in gut and soil microbiomes is well-established^5^. The condensed tannins, or proanthocyanidins, comprise flavan-3-ol subunits connected by chemically stable interflavan bonds that are degraded most conveniently with strong acid under oxidizing conditions^78^. Because condensed tannin (CT) appears to be more recalcitrant to degradation under biological conditions, it is an excellent substrate for this proof-of-concept study. Sorghum grain is a unique source for easily purifying hundreds of mg of CT as a chemically homogeneous preparation with a simple structure suitable for detailed metabolomic tracing.

Mature grain from high tannin *Sorghum bicolor* (L.) Moench grain (Hi-Tannin Sumac NM03-9905, Scott Bean, USDA Manhattan Kansas) was stored at 4 °C. Tannin was extracted from ground grain with methanol containing ascorbic acid and purified by ethyl acetate extraction to remove small phenolics, followed by Sephadex LH20 chromatography to isolate the high molecular weight fraction^21,79^. The freeze-dried powder was stored at −20 °C. The tannin was characterized by thiolysis to establish that the average degree of polymerization was 16, with a catechin terminal unit and epicatechin extenders (Fig. 1b). The material’s purity was assessed with NMR and HPLC (Supplementary Fig. 5, Supplementary Note 5)^21^.

### Reactor design and set up

To establish microcosms, frozen soil was thawed at room temperature for 1 h. 5 g of soil and the headspace was degassed in a Wheaton serum bottle for 30 min with 5 psi of N_2_ gas. A slurry was prepared by anoxically-transferring 125 mL anoxic sterile water via N_2_-degassed, sterile syringe to the degassed soil-containing serum bottle sealed with a butyl rubber stopper and an aluminum crimp. After inoculating the biologically active reactors with soil slurry (both CT amended and unamended), as discussed below, the remaining soil slurry was autoclaved three-times for 30 min each, and then inoculated into reactors as in the live controls. We confirmed we could not recover DNA or amplify DNA from the reactors inoculated with autoclaved soil slurry at each timepoint the biologically active samples were taken (Supplementary Note 1), supporting their microbially inactive status.

Anoxic reactors were established and sampled using prior methods that were demonstrated to support the growth of obligatory anaerobic metabolisms in soils and subsurface samples^20,80–82^. The medium was basal bicarbonate-buffered^20^, consisting of (per liter): 0.25 g ammonium chloride, 0.60 g sodium phosphate, 0.10 potassium chloride, 2.5 g sodium bicarbonate, 10 ml DL-vitamin mixture (Supplementary Table 2), and 10 ml DL-mineral mixture^83^ (Supplementary Table 2), and was brought to a pH of 7.0 using 1 mM NaOH. The biotic and autoclaved reactors were prepared with 90 mL and 45 mL, respectively, of media in 200 ml serum bottles with a N_2_-CO_2_ (80:20) headspace using standard anaerobic microbiology practices^80,84^. The anoxic soil slurry (autoclaved or biotic) was added to the reactors in a 1:10 dilution. CT-amended reactors (autoclaved and biotic) were established by adding anoxic, sterile CT stock solution in DI water (15 mg/ml), to achieve a final dosing of 1.5 mg/ml reactor. Reactors were flushed with N_2_-CO_2_ (80:20) gas in media-soil slurry and serum bottle head space for 40 min to ensure removal of trace oxygen before incubation.

Reactors were incubated in the dark and at 25 °C, consistent with field soil temperatures^20^. Here we selected field-relevant temperate operation (25 °C)^20^ to remove kinetic constraints on polyphenolic microbial growth and enzyme activity that were previously indicated in low temperature studies from boreal peatland soils^7,12,19^, as we consider it possible this temperature stress may have confounded interpretations of microbial polyphenol metabolism. Subsamples were collected over 20-days for 16S rRNA gene, metagenomic, metaproteomic, and various metabolomic and geochemical analyses (Fig. 1c). All subsamples were collected with care for maintaining anoxic conditions according to standard anaerobic microbiology protocols^80,82,84^, briefly, sampling was performed using sterile syringes that were degassed completely with N_2_-CO_2_ (80:20, vol/vol) to ensure no oxygen transfer. Subsamples were immediately dispensed into their respective storage tubes, flash frozen, and stored at −80 °C until processing/analysis.

Methane production was measured after 20-days as in Narrowe et al^20^. Briefly, we used a Shimadzu (GC-2014) gas chromatograph (GC) equipped with a thermal conductivity detector and using helium as a carrier gas at 100 °C to quantify methane from triplicate CT-amended and unamended control microcosms reactors at day 0 and at day 20.

### 16S rRNA Gene analyses

Total nucleic acids were extracted from the microcosms at days 0, 1, 3, 5, 7, 10, 14, and 20 using the Qiagen DNeasy PowerSoil Kit, and were stored at −20 °C until sequencing. Sequencing of the V4 region of the 16S rRNA gene was performed at Argonne National Laboratory’s Next Generation Sequencing Facility on the Illumina MiSeq using 251-bp paired-end reads and the Earth Microbiome Project primers (Supplementary Table 3)^85^. Reads were demultiplexed and analyzed within QIIME2 (2017.10) using DADA2^86^ to produce an amplicon sequence variant (ASV) by sample table (Supplementary Data 4), with taxonomy assigned using SILVA classifier (silva132.250). We filtered the feature table to contain only ASV’s observed in at least 3 samples. To survey ASV in reference databases, we BLASTed ASVs against RefSoil cultivated isolate genomes (of which 96% (*n* = 882) encode a 16S rRNA gene)^44^. ASV sequences were considered positive hits if they matched a sequence at greater than 97% identity over at least 74 bp (Supplementary Table 1).

### Metagenomic sequence and assembly

For days 5, 10, and 20, we obtained a CT- and control microcosm metagenome from pooled triplicate samples (*n* = 6 metagenomes). For this, genomic DNA was prepared for metagenomic sequencing using the Nextera XT Low Input-Illumina library creation kit, and was sequenced at the Department of Energy Joint Genome Institute on the Illumina NovaSeq 6000. Fastq files were trimmed using Sickle (v 1.33)^87^, and trimmed reads were assembled using IDBA-UD^88^ using k-mers (40, 60, 80, and 100). To maximize assembly, we performed (1) subtractive assemblies, iteratively assembling reads that did not map to assembled scaffolds ≥3 kb at 97% identity on all metagenomes, and (2) subassemblies using 25% of the combined CT-amended metagenome trimmed reads. Information for metagenome statistics, including assembly information, are found in Supplementary Table 2. For each assembly, scaffolds ≥2.5 kb were binned using MetaBAT2^89^ (v2.12.1), and MAG completion was assessed using AMPHORA2^90^ and checkM^91^ (v1.1.2). MAGs were kept in the database if they were >50% complete and <10% contaminated by either of these tools, or if it was >35% complete with <1% contamination in the event they recruited peptides in metaproteomes. MAGs were dereplicated at 99% identity using dRep^92^ (v2.6.2). MAG taxonomy was assigned using GTDB-tk (v1.3.0) R05-RS95^43^. See Supplementary Data 2 for MAG quality and taxonomy information.

MAGs and assemblies were annotated using DRAM^93^. CAZymes were inferred from the DRAM hits. Enzymes in Fig. 6c (except PGR) were mined from DRAM *raw* outputs. To mine C_15_ flavonoid enzymes (Fig. 6b and PGR), we constructed a custom database using published, characterized proteins^6,36,59,60^ (Supplementary Data 5). Using BLASTp, we searched for these enzymes in the metaproteome and in MAGs and putative hits were identified using a bit score cutoff greater than 150. Blast hits that met this criterion were further structurally modeled using PHYRE2^94^ web server to support putative roles. See Supplementary Fig. 16, Supplementary Data 3, and Supplementary Data 6 for structural modeling and BLASTp information, and sequences.

To quantify MAG relative abundance in each temporal sample and condition, trimmed metagenomic reads were mapped to the dereplicated MAG set using bbmap^95^ (v38.70) at minid=95, and output as sam files which were converted to sorted bam files using samtools^96^ (v1.9). We had two requirements for a MAG to be found in a sample: first we required reads to map to at least 75% of a MAG in a given sample, and second the MAG had to have at least 3X coverage in that sample. To determine MAGs that had reads mapped to at least 75% of the MAG, we used CoverM^97^ (v0.3.2) in genome mode to output MAGs that passed this threshold (–min-covered-fraction 75). To obtain MAG coverage, we used CoverM^97^ (v0.3.2) in genome mode to output reads_per_base (reads mapped/genome length), and from this calculated MAG coverage as reads_per_base x 151 bp. A bin was “present” in CT or in control if it was found with at least 3X average coverage across the MAG and had reads mapped to at least 75% of the MAG in any of the timepoints, or was “present” in both treatments if these two criteria were met in both CT and control metagenomes (ex. Present at day 5 in CT and at day 5 in Unamended). This information is given in Supplementary Data 2.

### Metaproteomic extraction and spectral analysis

Liquid culture (5 ml) from each microcosm sample was collected anaerobically, centrifuged for 15 min at 10,000 ×g, separated from the supernatant that was used for metabolite characterization and stored at −80 °C until shipment to Pacific Northwest National Laboratory. Proteins in the pellet were precipitated and washed twice with acetone. Then the pellet was lightly dried under nitrogen. 200 µl of an 8 M urea solution was added to the protein pellet, vortexed into solution. A bicinchoninic acid (BCA) assay (Thermo Scientific, Waltham, MA USA) was performed to determine protein concentration. Following the assay, 10 mM dithiothreitol (DTT) was added to the samples and incubated at 60 °C for 30 min with constant shaking at 2,552 xg. Samples were then diluted 8-fold for preparation for digestion with 100 mM NH4HCO3, 1 mM CaCl2 and sequencing-grade modified porcine trypsin (Promega, Madison, WI) was added to all protein samples at a 1:50 (w/w) trypsin-to-protein ratio for 3 h at 37 °C. Digested samples were desalted using a 4-probe positive pressure Gilson GX-274 ASPEC™ system (Gilson Inc., Middleton, WI) with Discovery C18 100 mg/1 ml solid phase extraction tubes (Supelco, St.Louis, MO), using the following protocol: 3 ml of methanol was added for conditioning followed by 2 mL of 0.1% TFA in H_2_O. The samples were then loaded onto each column followed by 4 ml of 95:5: H2O:ACN, 0.1% TFA. Samples were eluted with 1 ml 80:20 ACN:H2O, 0.1% TFA. The samples were concentrated down to ~30 µl using a Speed Vac and a final BCA was performed to determine the peptide concentration and samples were diluted to 0.1 µg/µl with nanopure water for MS analysis.

All mass-spectrometric data were acquired using an Orbitrap Lumos (Thermo Scientific) connected to a nanoACQUITY UPLC M-Class liquid chromatography system (Waters) via in-house 30-CM x 75-uM column packed using Reprocil-pur 1.9-μm C18 particles (Dr. Maisch HPLC GmbH, Germany) and in-house built electrospray apparatus. MS/MS spectra were compared with the custom metagenome and MAG database using the search tool MS-GF+^98^. Contaminant proteins typically observed in proteomics experiments were also included in the protein collections searched. The searches were performed using ±15-ppm parent mass tolerance, parent signal isotope correction, partially tryptic enzymatic cleavage rules, and variable oxidation of methionine. In addition, a decoy sequence approach was employed to assess false-discovery rates. Data were collated using an in-house program, imported into a SQL server database, filtered to ∼1% false-discovery rate (peptide to spectrum level), and combined at the protein level to provide (i) unique peptide count (per protein) and (ii) observation count (spectral count) data. We required at least two unique peptides per protein for identification, and for analyses used spectral counts from these identified proteins to calculate normalized spectral abundance factor (see below). See Supplementary Data 3.

### Metaproteomic database creation and analyses

The database for our metaproteome analysis was constructed from a dereplicated (100% amino acid identity) set of genes that were identified on binned and unbinned metagenomic scaffolds (i.e. all scaffolds >2.5 kb) (Fig. 4B). The inclusion of unbinned genes was done to allow us to account for assembled, expressed genes that were not assigned to genomic bins. We verified this Dereplicated Gene Database equally recruited metagenome reads from CT amended and CT unamended reactors, and thus was not biased by treatment (Supplementary Fig. 9, Supplementary Note 3). The CT-amended and unamended metaproteomes were mapped to this same Dereplicated Gene Database (Fig. 4d).

When reporting proteins identified in our metaproteome data, we assigned protein hits from our Dereplicated Gene Database to three categories (Fig. 4d). The first status was reported as “Non-Unique” if peptides identified from the mass spectra were assigned to in silico peptides that mapped to multiple genes in our Dereplicated Gene Database. The second status was reported as “unbinned unique” if peptides identified from the mass spectra were assigned to in silico peptides that mapped to a single gene, but this gene was not assigned to one of the reconstructed MAGs and was only assigned to an unbinned assembled scaffold. The third status was “binned unique”, where peptides identified from the mass spectra were assigned to in silico peptides that mapped to a single gene that was contained within a binned genome from our MAG database.

The three-classification system used in this metaproteomic analysis was designed to maximize the reporting of any expressed genes in a complex microbial community like soils, while also conservatively assigning gene expression to a specific genome where appropriate. The non-unique classification accounted for strain heterogeneity in soils with (i.e. several near identical genes in our database come from very closely related organisms and equally recruit peptides) and for proteins that have highly conserved sequences (i.e. ATP synthase). The expression patterns of these genes would have been excluded from downstream analyses if we relied only on unique peptide recovery. The unbinned-unique classification accounted for the fact some of the genes in our Dereplicated Gene Database were from assembled scaffolds that could not be assigned to a MAG through the genome binning process. The analyses reported in the manuscript used the binned-unique data (unless noted), with all reported proteome classification data shown in the supplementary analyses (Supplementary Fig. 11, Supplementary Data 3).

We took an untargeted, discovery-based approach to our metaproteomes and used label-free quantitation, consistent with many metaproteomic studies in environmental microbiomes to date^99–102^. Specifically, we used spectral counts where the number of unique spectra recovered for peptides are assumed to scale with their abundance. However, spectral counts are imperfect as they are biased by protein size and by sample-to-sample variation^103^ (Fig. 4e). Therefore, we converted spectral counts to normalized spectral abundance factor (NSAF), which includes normalizations that account for spectral count bias, making it a preferred method of quantitation from untargeted metaproteomes^103–106^. To calculate NSAF, the spectral count of a protein is divided by the protein length to give protein spectral abundance. This value is then divided by the sum of all protein spectral abundances to give the normalized spectral abundance^105^. This enabled comparison of a protein’s relative abundance within and across samples.

### Integrated metabolomic approaches

Historically, microbial transformations of polyphenols were inferred using low-resolution assays for total polyphenol content (i.e. the Folin–Ciocalteu assay) or CT-specific assays (ie. the acid butanol assay)^12^. Results from these assays have been the basis for theories like the “enzyme latch”, enabling the persistent idea that polyphenols are not susceptible to degradation under anoxic conditions^9^. However, these assays are not suitable for quantifying polyphenol content broadly in soils and especially for detailing the effects of microbial degradation of polyphenols in soils. For example, the widely-used Folin–Ciocalteu assay has limited quantitative application^26,27^ as it is nonspecific for quantifying polyphenols in complex matrices like soils, as the reagents react with a wide variety of compounds (e.g. thiols, vitamins, proteins, and inorganics^26^) contained within the soil matrix, thereby giving error prone concentrations of bulk polyphenols. Furthermore, polyphenols are structurally diverse, and “total polyphenol” content gives little information on structural changes. Additionally, the acid butanol assay for determining CT concentrations was shown to be non-specific for differentiating oligomer sizes of polyphenols^30^, meaning it would not resolve microbial depolymerization of the parent polyphenol into oligomers, a process which is a key indicator of degradation of condensed tannnins^5^. Further complicating the scenario, CT is highly reactive with protein biomass and soil matrix^22^, thus it is difficult to differentiate removal of CT by sorption and loss of CT due to biotransformation by microbes^28,29^. Therefore, we used high resolution instrumental approaches instead of chemical assays to identify metabolites indicative of (i) increased polymer depolymerization (breakdown into smaller oligomers and monomers) over time and (ii) production of further phenolic degradation metabolites.

To determine depolymerization of CT over time and the chemical degradation produced from microbial processes, we integrated metabolite data from several analytical techniques. Using this data, we specifically looked for metabolite evidence of the following fates for the added CT: depolymerization, here defined as breakage of the interflavan bond (Fig. 2a), biodegradation, here defined as signals that were unique to biologically-active soils relative to autoclaved soil, and transformation, here defined as signals that were temporally-distinct but could not be differentiated between biologically-active and autoclaved soils. Furthermore, we used this metabolite data to support other metabolisms happening in the reactors.

### FT-ICRMS analysis

We had two goals with our FTICR-MS analysis: (i) monitor changes in the CT polymer over time and (Supplementary Fig. 3a, Fig. 2) (ii) monitor changes in biochemical classes over time (Supplementary Fig. 3a, Fig. 7). Fourier Transform Ion Cyclotron resonance mass spectrometry (FTICR-MS) was used to collect high resolution mass spectra of the supernatant samples from reactors (microcosms) by direct injection in negative ion mode (Supplementary Note 6). For peaks that could be attributed to the CT polymer (Supplementary Fig. 3, Supplementary Note 6), Kendrick mass defect (KMD) analysis^107^ was then used to compare the fate of (epi)catechin CT oligomers over time in both biologically active and inactive (autoclaved soil) reactors. We used a modified version of KMD commonly used for polymer ions, proposed by Sato et al^32,108^, calculated using Eqs. 1–3.

1 $${\rm{KM}}({\rm{ion}})={\rm{m}}/{\rm{z}}({\rm{ion}})\ast (290/290.079038)$$

2 $${\rm{NM}}\_{\rm{CAT}}({\rm{ion}})={\rm{roundup}}({\rm{KM}}({\rm{ion}}))$$

3 $${\rm{KMD}}({\rm{ion}})={\rm{NM}}\_{\rm{CAT}}({\rm{ion}})-{\rm{KM}}({\rm{ion}})$$

To track changes in biochemical classes over time, putative chemical formulas of all peaks were assigned using Formularity (v1.0.0) software^109^ (Supplementary Fig. 3). Biochemical compound classes were reported as relative abundance values based on counts of C, H, and O for the following H:C and O:C ranges as in Tfaily et al.^110^. For more detailed information on FTICR-MS methodology and analyses, see Supplementary Note 6. Processed data is provided in Supplementary Data 7, and raw data provided in archive (doi:10.5281/zenodo.4552584.).

### LC–MS metabolomic analysis

Liquid chromatography-tandem mass spectrometry (LC–MS/MS) was used to identify exometabolites across samples over time. Metabolites were extracted into ethyl acetate from filtered supernatant samples after acidification with HCl. Both the aqueous and organic phases were dried down, redissolved, and analyzed by LC–MS/MS (Supplementary Note 7) using an Agilent 1290 UHPLC system connected to a Thermo Q Exactive Hybrid Quadrupole-Orbitrap Mass Spectrometer equipped with a Heated Electrospray Ionization (HESI-II) source probe. Separation, ionization, fragmentation and data acquisition parameters are specified in Supplementary Data 1. Briefly, metabolites were separated by gradient elution followed by MS1 and data dependent (top 2 most abundant MS1 ions not previously fragmented in last 7 s) MS2 collection; targeted data analysis was performed by comparison of sample peaks to a library of analytical standards analyzed under the same conditions. Three parameters were compared: matching m/z, retention time and fragmentation spectra using Metabolite Atlas (https://github.com/biorack/metatlas)^111,112^. Additional methodological details, including LC–MS parameters and MS resolution, are provided in Supplementary Data 1. Identification and standard reference comparison details are provided in Supplementary Data 1. For more information on LC–MS analyses, see Supplementary Note 7. To determine significantly discriminating LC–MS exometabolites, we applied a linear model to the log2-transformed peak area data using limma^113^ (v3.42.2) in R on log2-transformed data to compare metabolites in live and autoclaved treatments at each timepoint. Limma statistics are given in Supplementary Data 1.

### NMR metabolomic analysis

To follow important organic acids, we used NMR on supernatant samples. Supernatant samples (180 µL) were combined with 2,2-dimethyl-2-silapentane-5-sulfonate-d_6_ (DSS-d_6_) in D_2_O (20 µL, 5 mM) and thoroughly mixed prior to transfer to 3 mm NMR tubes. NMR spectra were acquired on a Varian 600 MHz VNMRS spectrometer equipped with a 5 mm triple-resonance (HCN) cold probe at a regulated temperature of 298 K. The 90° ^1^H pulse was calibrated prior to the measurement of each sample. The one-dimensional ^1^H spectra were acquired using a nuclear Overhauser effect spectroscopy (NOESY) pulse sequence with a spectral width of 12 ppm and 512 transients. The NOESY mixing time was 100 ms and the acquisition time was 4 s followed by a relaxation delay of 1.5 s during which presaturation of the water signal was applied. Time domain free induction decays (57472 total points) were zero filled to 131072 total points prior to Fourier transform. Chemical shifts were referenced to the ^1^H methyl signal in DSS-d_6_ at 0 ppm. The 1D ^1^H spectra were manually processed, assigned metabolite identifications and quantified using Chenomx NMR Suite 8.3. Metabolite identification was based on matching the chemical shift, J-coupling and intensity of experimental signals to compound signals in the Chenomx and custom in-house databases. Quantification was based on fitted metabolite signals relative to the internal standard (DSS-d_6_). Signal to noise ratios (S/N) were measured using MestReNova 14 with the limit of quantification equal to a S/N of 10 and the limit of detection equal to a S/N of 3. Processed data is available in Supplementary Data 1, and raw data provided in archive (doi:10.5281/zenodo.4552584).

### Reporting summary

Further information on research design is available in the Nature Research Reporting Summary linked to this article.

## Supplementary information

Supplementary Information

Description of Additional Supplementary Files

Supplementary Data 1

Supplementary Data 2

Supplementary Data 3

Supplementary Data 4

Supplementary Data 5

Supplementary Data 6

Supplementary Data 7

Reporting Summary

## Data Availability

The MAGs, assemblies, and reads resolved from the microcosm dataset reported in this paper have been deposited in National Center for Biotechnology Information BioProject PRJNA638681 (for biosample accession numbers see Supplementary Data 2). The RefSoil database used in this study can be found in the following figshare repository (https://figshare.com/articles/dataset/RefSoil_Database/4362812). The metaproteomics database queried in this study can be found in the following Zenodo archive with the identifier doi: 10.5281/zenodo.4578501. The mass spectrometry proteomics data have been deposited to the ProteomeXchange Consortium via the PRIDE^114^ partner repository with the dataset identifier PXD019911 (10.6019/PXD019911). FT-ICRMS and NMR data have been deposited in the following Zenodo archive with identifier doi:10.5281/zenodo.4552584. LC–MS data are available for download at the JGI Joint Genome Portal under ID 1281268 (https://genome.jgi.doe.gov/portal/202Coltabolomics_FD/202Coltabolomics_FD.info.html).

## References

[1] Li AN (2014). Resources and biological activities of natural polyphenols. Nutrients.

[2] Hättenschwiler S, Vitousek PM (2000). The role of polyphenols in terrestrial ecosystem nutrient cycling. Trends Ecol. Evol..

[3] Williamson G (2017). The role of polyphenols in modern nutrition. Nutr. Bull..

[4] Correddu F (2020). Can agro-industrial by-products rich in polyphenols be advantageously used in the feeding and nutrition of dairy small ruminants?. Animals.

[5] Bhat TK, Singh B, Sharma OP (1998). Microbial degradation of tannins - a current perspective. Biodegradation.

[6] Braune A, Blaut M (2016). Bacterial species involved in the conversion of dietary flavonoids in the human gut. Gut Microbes.

[7] D Zak (2019). Unraveling the importance of polyphenols for microbial carbon mineralization in rewetted riparian peatlands. Front. Environ. Sci.

[8] Fenner N, Freeman C (2011). Drought-induced carbon loss in peatlands. Nat. Geosci..

[9] Freeman C, Ostle N, Kang H (2001). An enzymic “latch” on a global carbon store: a shortage of oxygen locks up carbon in peatlands by restraining a single enzymes. Nature.

[10] Fenner N, Freeman C (2020). Woody litter protects peat carbon stocks during drought. Nat. Clim. Chang..

[11] Alshehri A (2020). A potential approach for enhancing carbon sequestration during peatland restoration using low-cost, phenolic-rich biomass supplements. Front. Environ. Sci..

[12] Fierer N, Schimel JP, Cates RG, Zou J (2001). Influence of balsam poplar tannin fractions on carbon and nitrogen dynamics in Alaskan taiga floodplain soils. Soil Biol. Biochem..

[13] Brouns K, Keuskamp JA, Potkamp G, Verhoeven JTA, Hefting MM (2016). Peat origin and land use effects on microbial activity, respiration dynamics and exo-enzyme activities in drained peat soils in the Netherlands. Soil Biol. Biochem..

[14] Bonnett SAF, Maltby E, Freeman C (2017). Hydrological legacy determines the type of enzyme inhibition in a peatlands chronosequence. Sci. Rep.

[15] Schmidt MA (2013). Soil microbial communities respond differently to three chemically defined polyphenols. Plant Physiol. Biochem..

[16] Swenson TL, Karaoz U, Swenson JM, Bowen BP, Northen TR (2018). Linking soil biology and chemistry in biological soil crust using isolate exometabolomics. Nat. Commun.

[17] Woodcroft BJ (2018). Genome-centric view of carbon processing in thawing permafrost. Nature.

[18] Angle JC (2017). Methanogenesis in oxygenated soils is a substantial fraction of wetland methane emissions. Nat. Commun..

[19] Pinsonneault AJ, Moore TR, Roulet NT (2016). Temperature the dominant control on the enzyme-latch across a range of temperate peatland types. Soil Biol. Biochem..

[20] Narrowe AB (2019). Uncovering the diversity and activity of methylotrophic methanogens in freshwater wetland soils. mSystems.

[21] Reeves, S. G. et al. Proanthocyanidin structural details revealed by ultrahigh resolution FT-ICR MALDI-mass spectrometry, 1H– 13 C HSQC NMR, and thiolysis-HPLC–DAD. *J. Agric. Food Chem*. (2020). 10.1021/acs.jafc.0c04877. 10.1021/acs.jafc.0c04877 33170695

[22] Kraus TEC, Dahlgren RA, Zasoski RJ (2003). Tannins in nutrient dynamics of forest ecosystems - a review. Plant Soil.

[23] Triebwasser DJ, Tharayil N, Preston CM, Gerard PD (2012). The susceptibility of soil enzymes to inhibition by leaf litter tannins is dependent on the tannin chemistry, enzyme class and vegetation history. N. Phytol..

[24] Halvorson JJ, Gonzalez JM, Hagerman AE (2011). Repeated applications of tannins and related phenolic compounds are retained by soil and affect cation exchange capacity. Soil Biol. Biochem..

[25] Buessecker S (2019). Effects of sterilization techniques on chemodenitrification and N2O production in tropical peat soil microcosms. Biogeosciences.

[26] Everette JD (2010). Thorough study of reactivity of various compound classes toward the folin-Ciocalteu reagent. J. Agric. Food Chem..

[27] Sánchez-Rangel JC, Benavides J, Heredia JB, Cisneros-Zevallos L, Jacobo-Velázquez DA (2013). The Folin-Ciocalteu assay revisited: Improvement of its specificity for total phenolic content determination. Anal. Methods.

[28] Schofield JA, Hagerman AE, Harold A (1998). Loss of tannins and other phenolics from: Willow leaf litter. J. Chem. Ecol..

[29] Adamczyk B, Kiikkilä O, Kitunen V, Smolander A (2014). Can we measure condensed tannins from tannin-protein complexes? - A case study with acid-butanol assay in boreal forest soil organic layer. Eur. J. Soil Biol..

[30] Li C, Trombley JD, Schmidt MA, Hagerman AE (2010). Preparation of an acid butanol standard from fresh apples. J. Chem. Ecol..

[31] Talbot JM, Finzi AC (2008). Differential effects of sugar maple, red oak, and hemlock tannins on carbon and nitrogen cycling in temperate forest soils. Oecologia.

[32] Fouquet TNJ (2018). On the Kendrick mass defect plots of multiply charged polymer ions: splits, misalignments, and how to correct them. J. Am. Soc. Mass Spectrom..

[33] Fouquet TNJ (2019). The Kendrick analysis for polymer mass spectrometry. J. Mass Spectrom..

[34] Wang CM, Li TC, Jhan YL, Weng JH, Chou CH (2013). The Impact of microbial biotransformation of catechin in enhancing the allelopathic effects of Rhododendron formosanum. PLoS One.

[35] Smeriglio A, Barreca D, Bellocco E, Trombetta D (2017). Proanthocyanidins and hydrolysable tannins: occurrence, dietary intake and pharmacological effects. Br. J. Pharmacol..

[36] Braune A, Gütschow M, Blauta M (2019). An NADH-dependent reductase from Eubacterium ramulus catalyzes the stereospecific heteroring cleavage of flavanones and flavanonols. Appl. Environ. Microbiol..

[37] Zhao H, Xu Y, Lin S, Spain JC, Zhou N-Y (2018). The molecular basis for the intramolecular migration (NIH shift) of the carboxyl group during *para* -hydroxybenzoate catabolism. Mol. Microbiol..

[38] Williamson G, Kay CD, Crozier A (2018). The bioavailability, transport, and bioactivity of dietary flavonoids: a review from a historical perspective. Compr. Rev. Food Sci. Food Saf..

[39] Monagas M (2010). Insights into the metabolism and microbial biotransformation of dietary flavan-3-ols and the bioactivity of their metabolites. Food Funct..

[40] Fierer N (2017). Embracing the unknown: disentangling the complexities of the soil microbiome. Nat. Rev. Microbiol..

[41] Bowers RM (2017). Minimum information about a single amplified genome (MISAG) and a metagenome-assembled genome (MIMAG) of bacteria and archaea. Nat. Biotechnol..

[42] Shade A (2014). Conditionally rare taxa disproportionately contribute to temporal changes in microbial diversity. MBio.

[43] Chaumeil PA, Mussig AJ, Hugenholtz P, Parks DH (2020). GTDB-Tk: A toolkit to classify genomes with the genome taxonomy database. Bioinformatics.

[44] Choi J (2017). Strategies to improve reference databases for soil microbiomes. ISME J..

[45] Arafat Y (2020). Soil sickness in aged tea plantation is associated with a shift in microbial communities as a result of plant polyphenol accumulation in the tea gardens. Front. Plant Sci..

[46] Roopchand DE (2015). Dietary polyphenols promote growth of the gut bacterium akkermansia muciniphila and attenuate high-fat diet-induced metabolic syndrome. Diabetes.

[47] Janusz G (2017). Lignin degradation: microorganisms, enzymes involved, genomes analysis and evolution. FEMS Microbiol. Rev..

[48] Kamimura N, Sakamoto S, Mitsuda N, Masai E, Kajita S (2019). Advances in microbial lignin degradation and its applications. Curr. Opin. Biotechnol..

[49] Orellana R (2017). Multi-time series RNA-seq analysis of Enterobacter lignolyticus SCF1 during growth in lignin-amended medium. PLoS ONE.

[50] DeAngelis KM (2013). Evidence supporting dissimilatory and assimilatory lignin degradation in Enterobacter lignolyticus SCF1. Front. Microbiol.

[51] Chen Y, Huang Z, Li J, Su G, Feng B (2020). Complete genome sequence of Kosakonia radicincitans GXGL-4A, a nitrogen-fixing bacterium with capability to degrade TEX. Curr. Microbiol..

[52] Pletzer D, Weingart H (2014). Characterization and regulation of the Resistance-Nodulation-Cell Division-type multidrug efflux pumps MdtABC and MdtUVW from the fire blight pathogen Erwinia amylovora. BMC Microbiol..

[53] Zoetendal EG, Smith AH, Sundset MA, Mackie RI (2008). The BaeSR two-component regulatory system mediates resistance to condensed tannins in Escherichia coli. Appl. Environ. Microbiol..

[54] Soares AR (2014). The role of L-DOPA in plants. Plant Signal. Behav..

[55] Rekdal CM, Bess EN, Bisanz EJ, Turnbaugh PJ, Balskus EP (2019). Discovery and inhibition of an interspecies gut bacterial pathway for Levodopa metabolism. Science.

[56] Aura AM (2013). Characterization of microbial metabolism of Syrah grape products in an in vitro colon model using targeted and non-targeted analytical approaches. Eur. J. Nutr..

[57] Li AN, Li AN (2019). The gut microbiota links dietary polyphenols with management of psychiatric mood disorders. Front. Neurosci..

[58] Braune A (2016). Chalcone isomerase from Eubacterium ramulus catalyzes the ring contraction of flavanonols. J. Bacteriol..

[59] Schoefer L, Braune A, Blaut M (2004). Cloning and expression of a phloretin hydrolase gene from Eubacterium ramulus and characterization of the recombinant enzyme. Appl. Environ. Microbiol..

[60] Han J (2019). Discovery and structural analysis of a phloretin hydrolase from the opportunistic human pathogen *Mycobacterium abscessus*. FEBS J..

[61] Haddock JD, Ferry JG (1989). Purification and properties of phloroglucinol reductase from Eubacterium oxidoreducens G-41. J. Biol. Chem..

[62] Ozawa Y (2012). Indolepyruvate ferredoxin oxidoreductase: An oxygen-sensitive iron-sulfur enzyme from the hyperthermophilic archaeon Thermococcus profundus. J. Biosci. Bioeng..

[63] Carmona M (2009). Anaerobic catabolism of aromatic compounds: a genetic and genomic view. Microbiol. Mol. Biol. Rev..

[64] Dilling S, Imkamp F, Schmidt S, Müller V (2007). Regulation of caffeate respiration in the acetogenic bacterium Acetobacterium woodii. Appl. Environ. Microbiol..

[65] Zhang Z (2019). Priming effects of soil organic matter decomposition with addition of different carbon substrates. J. Soils Sediment..

[66] Brune A, Schnell S, Schinkt B (1992). Sequential transhydroxylations converting hydroxyhydroquinone to phloroglucinol in the strictly anaerobic, fermentative bacterium pelobacter massiliensis. Appl. Environ. Microbiol..

[67] Vasta V (2019). Plant polyphenols and rumen microbiota responsible for fatty acid biohydrogenation, fiber digestion, and methane emission: Experimental evidence and methodological approaches. J. Dairy Sci..

[68] Kato S (2015). Methanogenic degradation of lignin-derived monoaromatic compounds by microbial enrichments from rice paddy field soil. Sci. Rep..

[69] Kuusk S (2018). Kinetics of H2O2-driven degradation of chitin by a bacterial lytic polysaccharide monooxygenase. J. Biol. Chem..

[70] Roy Chowdhury T (2019). Metaphenomic responses of a native prairie soil microbiome to moisture perturbations. mSystems.

[71] Wilhelm RC, Singh R, Eltis LD, Mohn WW (2019). Bacterial contributions to delignification and lignocellulose degradation in forest soils with metagenomic and quantitative stable isotope probing. ISME J..

[72] Hatzenpichler R (2016). Visualizing in situ translational activity for identifying and sorting slow-growing archaeal - bacterial consortia. Proc. Natl Acad. Sci. USA.

[73] Pett-Ridge J, Weber PK (2012). NanoSIP: NanoSIMS applications for microbial biology. Methods Mol. Biol..

[74] Bailey VL (2013). Micrometer-scale physical structure and microbial composition of soil macroaggregates. Soil Biol. Biochem..

[75] Min K, Freeman C, Kang H, Choi SU (2015). The regulation by phenolic compounds of soil organic matter dynamics under a changing environment.. Biomed Res. Int.

[76] Quideau S, Deffieux D, Douat-Casassus C, Pouységu L (2011). Plant polyphenols: chemical properties, biological activities, and synthesis. Angew. Chem. - Int. Ed..

[77] Hagerman, A. E. Fifty Years of Polyphenol-Protein Complexes. In *Recent Advances in Polyphenol Research* **3**, 71–97 (Wiley-Blackwell, 2012).

[78] Porter LJ, Hrstich LN, Chan BG (1985). The conversion of procyanidins and prodelphinidins to cyanidin and delphinidin. Phytochemistry.

[79] Tannin Handbook - Ann E. Hagerman. Available at: http://www.users.miamioh.edu/hagermae/.

[80] Wrighton KC (2010). Bacterial community structure corresponds to performance during cathodic nitrate reduction. ISME J..

[81] van Trump Ian JIJ (2011). Humic acid-oxidizing, nitrate-reducing bacteria in agricultural soils. MBio.

[82] Borton MA (2018). Coupled laboratory and field investigations resolve microbial interactions that underpin persistence in hydraulically fractured shales. Proc. Natl Acad. Sci. USA.

[83] Lovley DR, Phillips EJP (1988). Novel mode of microbial energy metabolism: organic carbon oxidation coupled to dissimilatory reduction of iron or manganese. Appl. Environ. Microbiol..

[84] Gregory KB, Bond DR, Lovley DR (2004). Graphite electrodes as electron donors for anaerobic respiration. Environ. Microbiol..

[85] Caporaso JG (2012). Ultra-high-throughput microbial community analysis on the Illumina HiSeq and MiSeq platforms. ISME J..

[86] Callahan BJ (2016). DADA2: High-resolution sample inference from Illumina amplicon data. Nat. Methods.

[87] Joshi, N. & Fass, J. Sickle: A sliding-window, adaptive, quality-based trimming tool for FastQ files (Version 1.33) [Software]. Available at https://github.com/najoshi/sickle. 2011 (2011).

[88] Peng Y, Leung HCM, Yiu SM, Chin FYL (2012). IDBA-UD: A de novo assembler for single-cell and metagenomic sequencing data with highly uneven depth. Bioinformatics.

[89] Kang DD (2019). MetaBAT 2: An adaptive binning algorithm for robust and efficient genome reconstruction from metagenome assemblies. PeerJ.

[90] Wu M, Scott AJ (2012). Phylogenomic analysis of bacterial and archaeal sequences with AMPHORA2. Bioinformatics.

[91] Parks DH, Imelfort M, Skennerton CT, Hugenholtz P, Tyson GW (2015). CheckM: assessing the quality of microbial genomes recovered from isolates, single cells, and metagenomes. Genome Res..

[92] Olm MR, Brown CT, Brooks B, Banfield JF (2017). DRep: A tool for fast and accurate genomic comparisons that enables improved genome recovery from metagenomes through de-replication. ISME J..

[93] Shaffer M (2020). DRAM for distilling microbial metabolism to automate the curation of microbiome function. Nucleic Acids Res..

[94] Kelley LA, Mezulis S, Yates CM, Wass MN, Sternberg MJE (2015). The Phyre2 web portal for protein modeling, prediction and analysis. Nat. Protoc..

[95] Bushnell, B. BBMap: A Fast, Accurate, Splice-Aware Aligner. (2014). Available at: sourceforge.net/projects/bbmap/.

[96] Li H (2009). The sequence alignment/map format and SAMtools. Bioinformatics.

[97] CoverM: Read coverage calculator for metagenomics. Available at: https://github.com/wwood/CoverM.

[98] Kim S, Pevzner PA (2014). MS-GF+ makes progress towards a universal database search tool for proteomics. Nat. Commun..

[99] Herold M (2020). Integration of time-series meta-omics data reveals how microbial ecosystems respond to disturbance. Nat. Commun.

[100] Diamond S (2019). Mediterranean grassland soil C–N compound turnover is dependent on rainfall and depth, and is mediated by genomically divergent microorganisms. Nat. Microbiol..

[101] Schneider T (2012). Who is who in litter decomposition Metaproteomics reveals major microbial players and their biogeochemical functions. ISME J..

[102] Hultman J (2015). Multi-omics of permafrost, active layer and thermokarst bog soil microbiomes. Nature.

[103] Von Bergen M (2013). Insights from quantitative metaproteomics and protein-stable isotope probing into microbial ecology. ISME J..

[104] Zorz JK (2019). A shared core microbiome in soda lakes separated by large distances. Nat. Commun..

[105] Hawley AK, Brewer HM, Norbeck AD, Paša-Tolić L, Hallam SJ (2014). Metaproteomics reveals differential modes of metabolic coupling among ubiquitous oxygen minimum zone microbes. Proc. Natl Acad. Sci. USA.

[106] Neilson KA, Keighley T, Pascovici D, Cooke B, Haynes PA (2013). Label-free quantitative shotgun proteomics using normalized spectral abundance factors. Methods Mol. Biol..

[107] Hughey CA, Hendrickson CL, Rodgers RP, Marshall AG, Qian K (2001). Kendrick mass defect spectrum: a compact visual analysis for ultrahigh-resolution broadband mass spectra. Anal. Chem..

[108] Sato H, Nakamura S, Teramoto K, Sato T (2014). Structural characterization of polymers by MALDI spiral-TOF mass spectrometry combined with kendrick mass defect analysis. J. Am. Soc. Mass Spectrom..

[109] Tolić N (2017). Formularity: software for automated formula assignment of natural and other organic matter from ultrahigh-resolution mass spectra. Anal. Chem..

[110] Tfaily MM (2017). Sequential extraction protocol for organic matter from soils and sediments using high resolution mass spectrometry. Anal. Chim. Acta.

[111] Yao Y (2015). Analysis of metabolomics datasets with high-performance computing and metabolite atlases. Metabolites.

[112] Bowen BP, Northen TR (2010). Dealing with the unknown: metabolomics and metabolite atlases. J. Am. Soc. Mass Spectrom..

[113] Smyth, G. K. Limma: linear models for microarray data. In *Bioinformatics and Computational Biology Solutions Using R and Bioconductor* 397–420 (2005).

[114] Perez-Riverol Y (2019). The PRIDE database and related tools and resources in 2019: improving support for quantification data. Nucleic Acids Res.

