# Supplementary material for: Decrypting bacterial polyphenol metabolism in an anoxic wetland soil
Source: Nat Commun. 2021 Apr 29;12:2466. doi: 10.1038/s41467-021-22765-1 (PMC8084988; doi:10.1038/s41467-021-22765-1)
Supplement: Supplementary file 1 — Supplementary Information [file 41467_2021_22765_MOESM1_ESM.pdf]

Supplementary Information for:

Decrypting bacterial polyphenol metabolism in an anoxic wetland soil

McGivern, et al.

Table of Contents

Supplementary Figures 1-16	2-20
Supplementary Table 1	20
Supplementary Table 2	21
Supplementary Table 3	22
Supplementary Notes 1-7	23-32
References	32-34

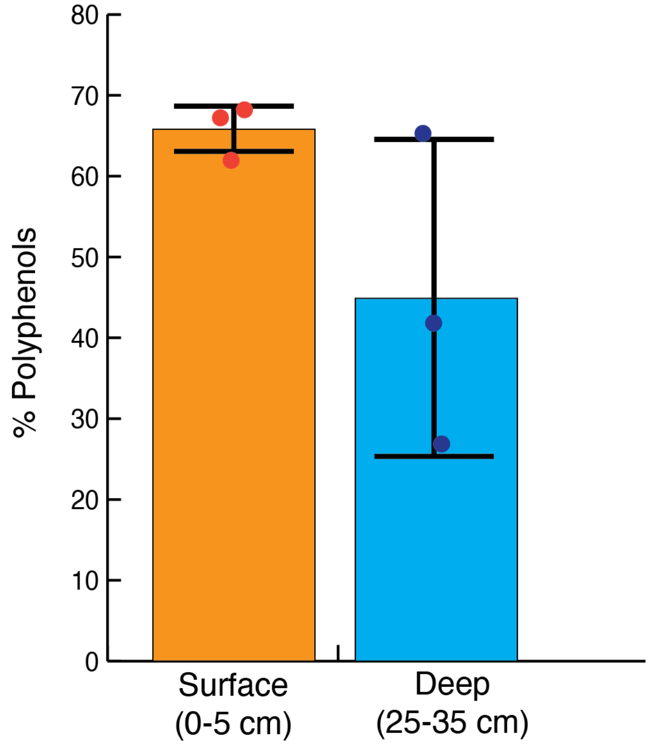

Supplementary Figure 1. Abundance of polyphenol-like compounds in field surface and deep wetland soils. Polyphenol-like compound abundance in Fourier-transform ion cyclotron resonance mass spectrometry (FTICR-MS) data from the wetland, which was used as the soil microbial community inoculum in our microcosms. Here we used the surface soil as our inoculum. Barplots of FTICR-MS identified average lignin- and tannin-like peak abundance in surface (orange) and deep (blue) soils, with individual datapoints overlaid. Error bars show one standard deviation (n=3 independent soil samples from each region).

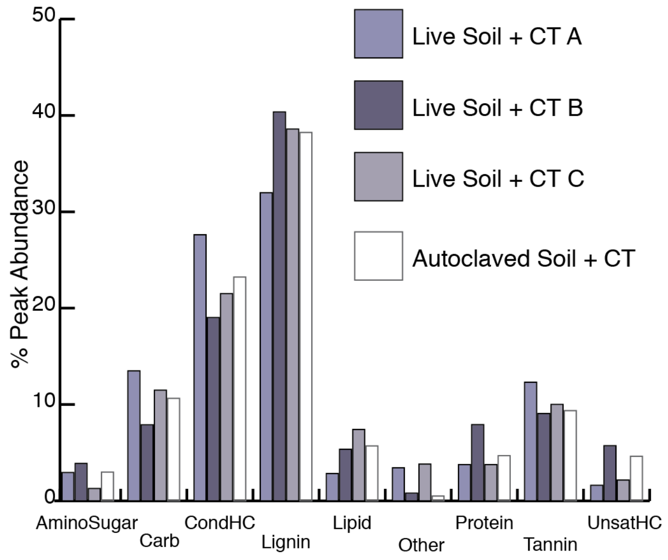

Supplementary Figure 2. Abundance of FTICR-MS identified biochemical classes between live and autoclaved soil samples. Peak abundance of biochemical classes identified in FTICR-MS across biotic CT replicate and autoclaved CT soil microcosms at day 0. Peaks were classified as described in **Materials and Methods**, and the relative abundance is shown across each biochemical class for each sample (n=1 each sample type). Raw data is provided in **Supplementary Data 7.**

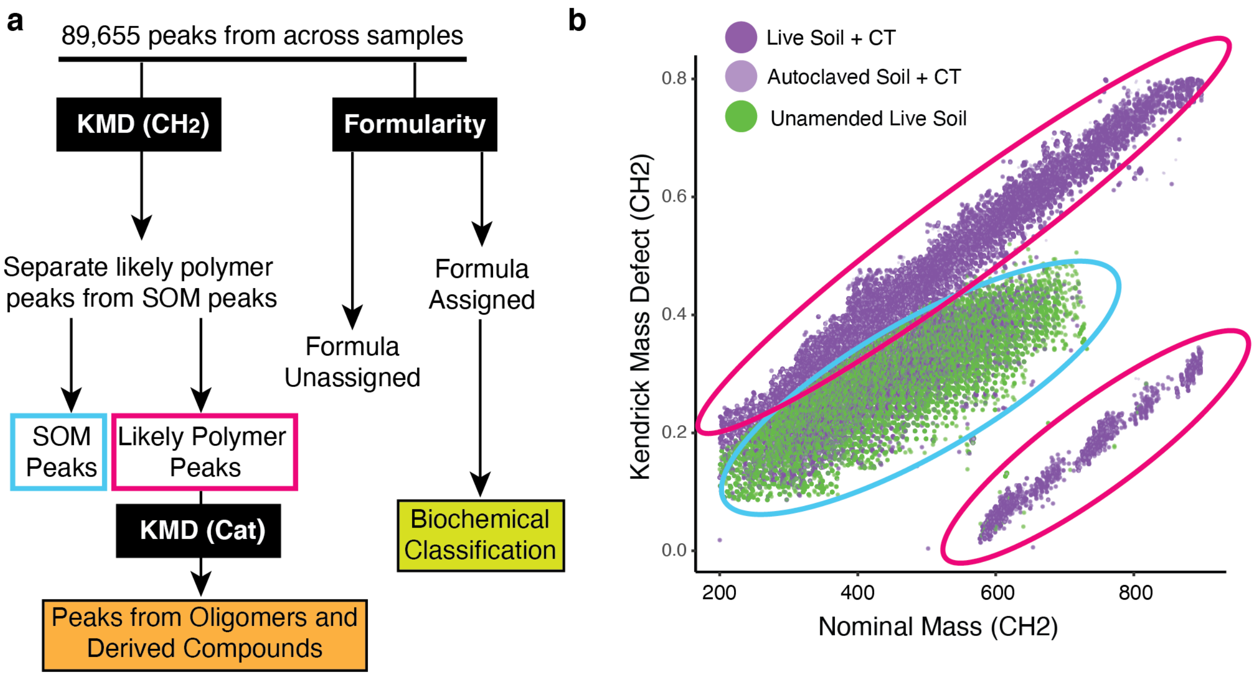

Supplementary Figure 3. FTICR-MS workflow and identification of CT-derived peaks across samples. (**a**) We had two goals for analyzing the FT-ICRMS data: (i) identify peaks that derived from CT polymer oligomers and derived compounds (orange box), and (ii) assign identified peaks to biochemical classes (green box). This workflow is described in **Supplementary Note 6.** (**b**) CT polymer peaks were identified across all identified peaks by Kendrick Mass defect analysis using CH_2 as the base unit (**Supplementary Note 6**), and comparison across CT-amended samples (purple) and unamended samples (green). Peaks that derived predominantly from CT-amended samples (pink ovals) were assumed to correspond to CT and CT-derived compounds while majority shared peaks (blue oval) were assumed to be soil organic matter (SOM) derived.

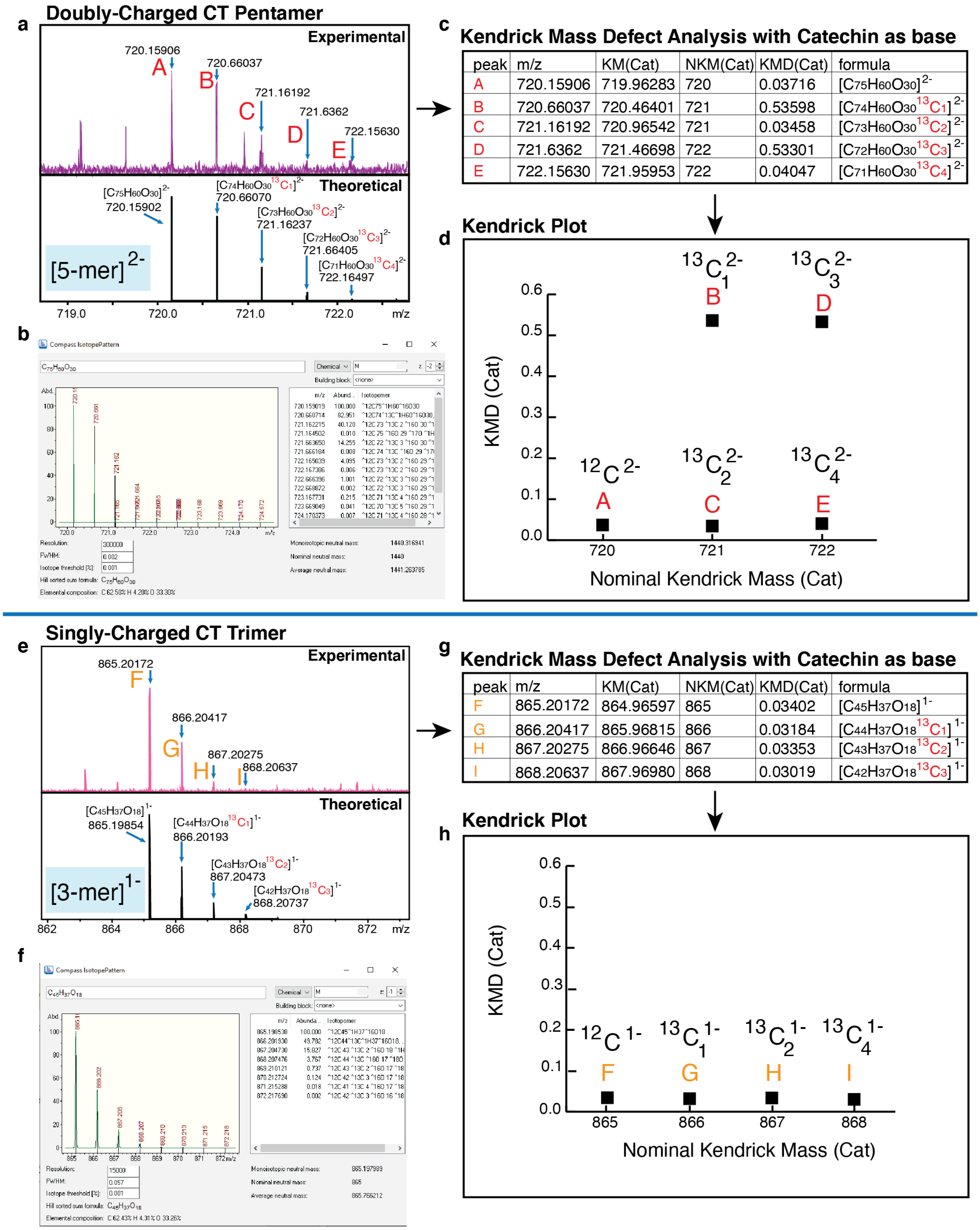

Supplementary Figure 4. Identification and example Kendrick Mass Defect analysis of variably-charged CT oligomers. (**a**) FTICR mass spectrum of a doubly-charged CT

pentamer in Live Soil +CT replicate A at day 10 (top) compared to the expected mass spectrum for a doubly-charged CT pentamer (bottom). Peak masses are indicated, and the formulas for peaks in the theoretical spectra are given. (**b**) The isotope abundances match expected abundances, showing Bruker software (**Supplementary Note 6**). (**c**) The five experimentally identified peaks (A-E) were used in KMD analysis using (epi)catechin as base, and (**d**) plotted in a Kendrick plot. (**e-h**) This same analysis was repeated with peaks corresponding to a singly charged CT trimer identified in Live Soil +CT replicate A at day 10. Comparison of the Kendrick plots for the doubly-charged (**d**) and singly-charged (**h**) isotopomers reveals isotopic splitting where KMD separates isotopes at 1/z as in Fouquet *et al*[1]. Abbreviations are as follows: Kendrick Mass (KM), Nominal Kendrick Mass (NKM), and Kendrick Mass Defect (KMD).

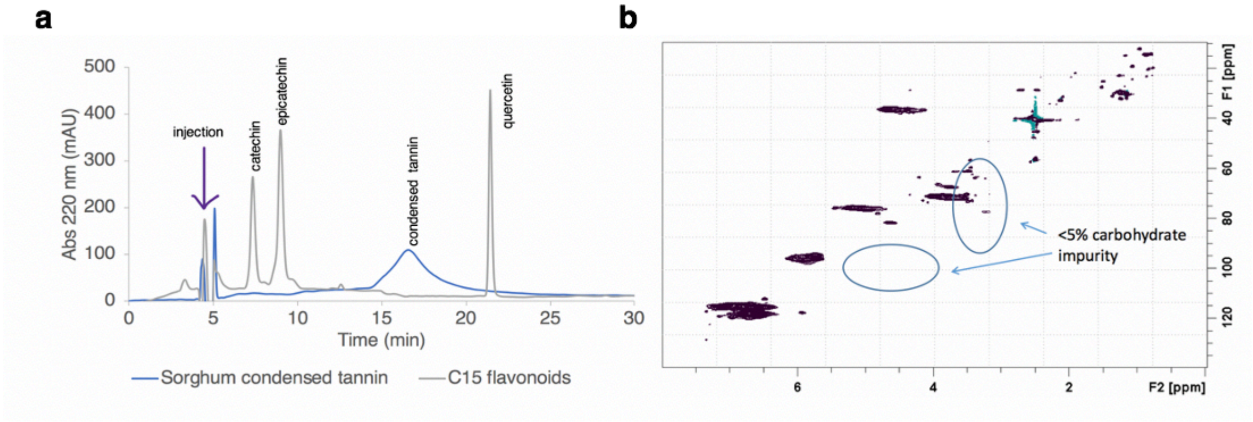

Supplementary Fig 5. Characterization of the CT substrate used in this study showed minimal contamination from phenolics or carbohydrates. (a) Overlaid chromatograms for a Sorghum condensed tannin purified sample (blue) and a mixture of commercial catechin, epicatechin and quercetin (grey). The Sorghum condensed tannin elutes as a broad peak with no resolution of individual polymers or oligomers. Here, we observed the CT contained no monomers. (**b**) A representative ^{1}H-^{13}C HSQC NMR spectrum of purified condensed tannin from Sorghum. The blue ovals represent the areas where carbohydrate signals are found. The signals in those regions are all assigned to condensed tannin structural features as detailed in Reeves *et al.*[2]. For chromatography and NMR methods, see **Supplementary Note 5**.

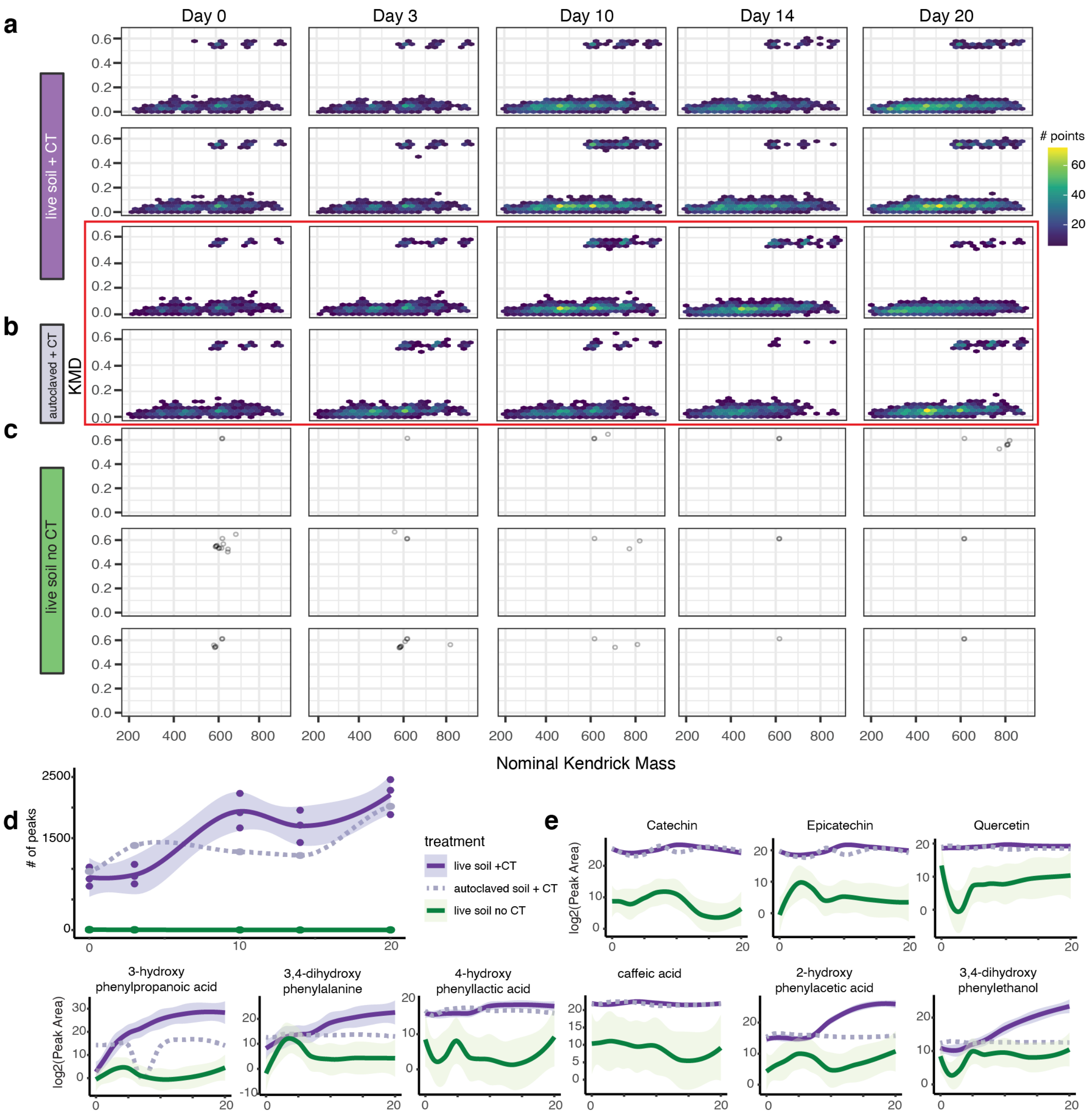

Supplementary Figure 6. **Unamended control soils do not contain appreciable CT oligomers or putative CT-degradation products.** Kendrick mass defect plots based on (epi)catechin as the repeat unit for (**a**) live soil +CT replicates, (**b**) autoclaved soil + CT control, and (**c**) unamended control soil. A red box is shown around the two plots shown in **Figure 2.** Hex plots are used in **a** and **b** because of the high number of data points, while individual points are plotted in **c**. Hex plots divide plot area into equal size hexagons, and hexagons are colored according to the number of data points that fall in that area. (**d**) The number of CT-associated peaks are shown with lines indicating average number of peaks for CT (purple, n=3 independent biological replicates) and unamended control (green, n=3 independent biological replicates) microcosms, and shaded areas the 95%

confidence interval. Dotted lines show peaks in autoclaved CT-amended soil control. (**e**) LC-MS identified compounds highlighted in **Figure 2** for all samples. Metabolite dynamics are shown with lines indicating average peak area (n=3 independent biological replicates) for CT (purple) and (n=3 independent biological replicates) unamended control (green) microcosms, and shaded areas the 95% confidence interval. Dotted lines show signal from autoclaved CT-amended soil controls. Raw Data is provided in **Supplementary Data 1.**

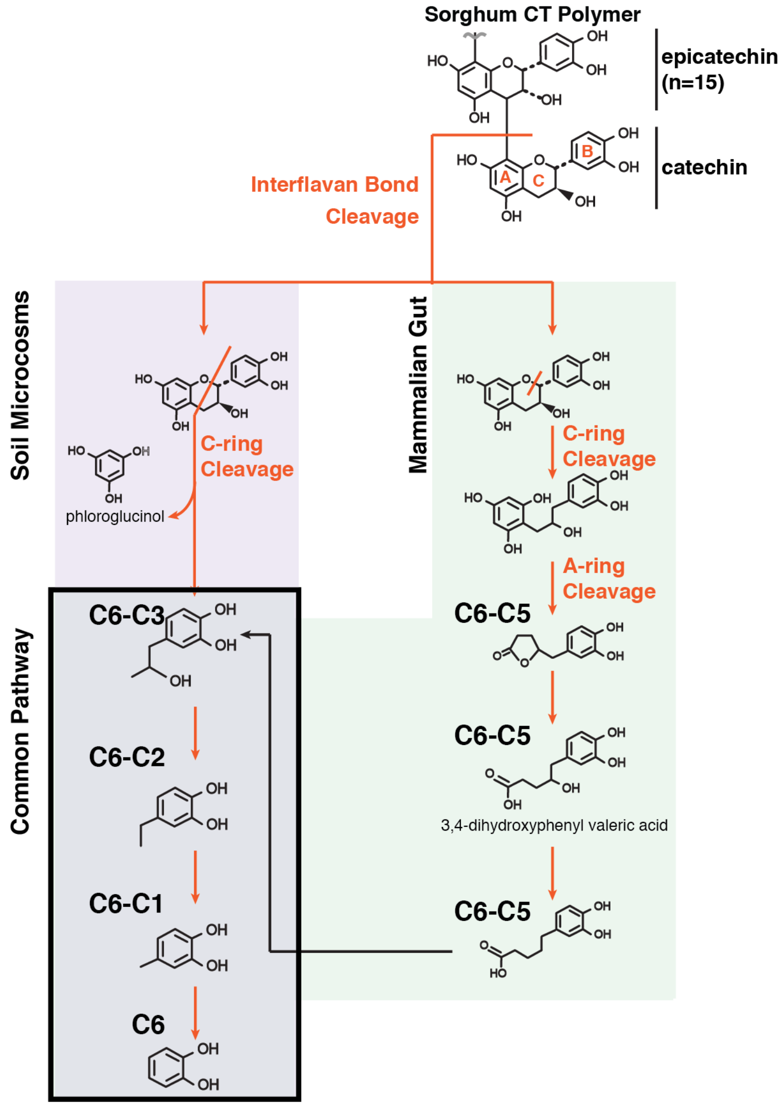

Supplementary Figure 7. Comparison of proposed CT degradation pathways between mammalian guts and soil from this study. Metabolomic evidence suggests differing strategies for initial steps in CT degradation between mammalian gut and soil systems. In our soil system, depolymerization is followed by a C-ring opening to yield the C_6-C_3 products characteristic of our reactors, while in the gut the products point to cleavage of the C-ring followed by opening of the A-ring to yield C_6-C_5 products. These C_6-C_5 products are suggested in gut studies to converge at C_6-C_3 products, suggesting some commonality in degradation across systems.

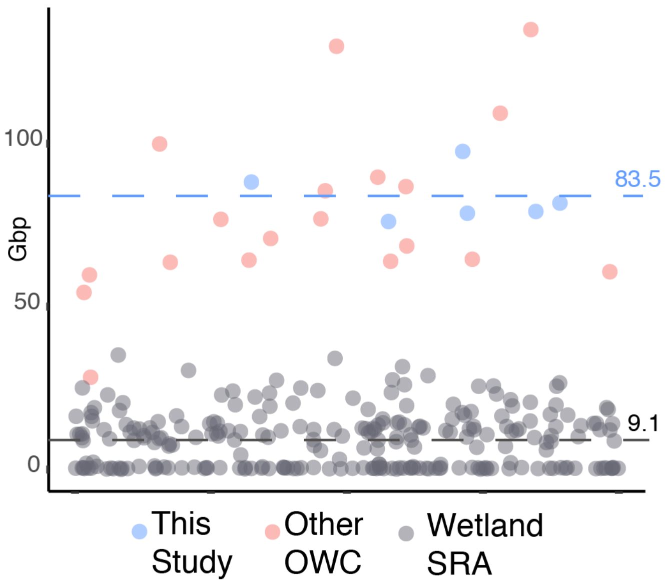

Supplementary Figure 8. The depth of metagenomic sequencing in this study exceeds standard sequencing depth for wetland soil metagenomes. Distribution in sequencing (as Gigabases) for Sequence Read Archive (SRA) runs identified as “WGS” with “Wetland” in the metadata. Samples from this study are shown in blue, while other studies from our lab conducted on Old Woman Creek (OWC) samples are shown in red. All other identified SRA runs are shown in grey. The blue and grey dotted lines and numbers at left show the average sequencing per sample for this study and other wetland SRA studies, respectively.

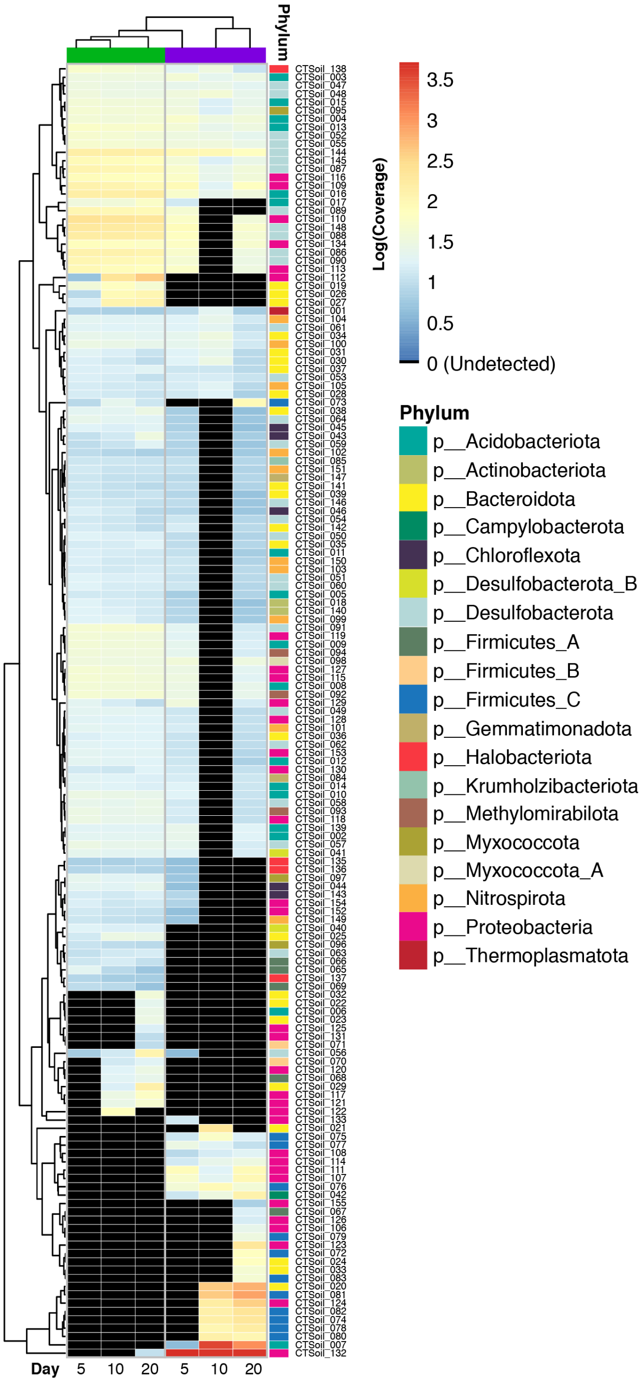

Supplementary Figure 9. Metagenome-assembled genome (MAG) detection across the six metagenomes revealed core and treatment specific MAGs. The coverage of the 155 dereplicated MAGs is shown across metagenomes. Bins highlighted in black did not meet “presence” requirement of 75% scaffolds mapped at 95% identity and greater than 3X coverage. Adjacent colored boxes indicate bin Phylum, with label showing bin number. The columns highlighted with green (left) are unamended controls, while the columns highlighted with purple (right) are from CT-amended samples. The same data is reported as presence and absence in **Figure 3**.

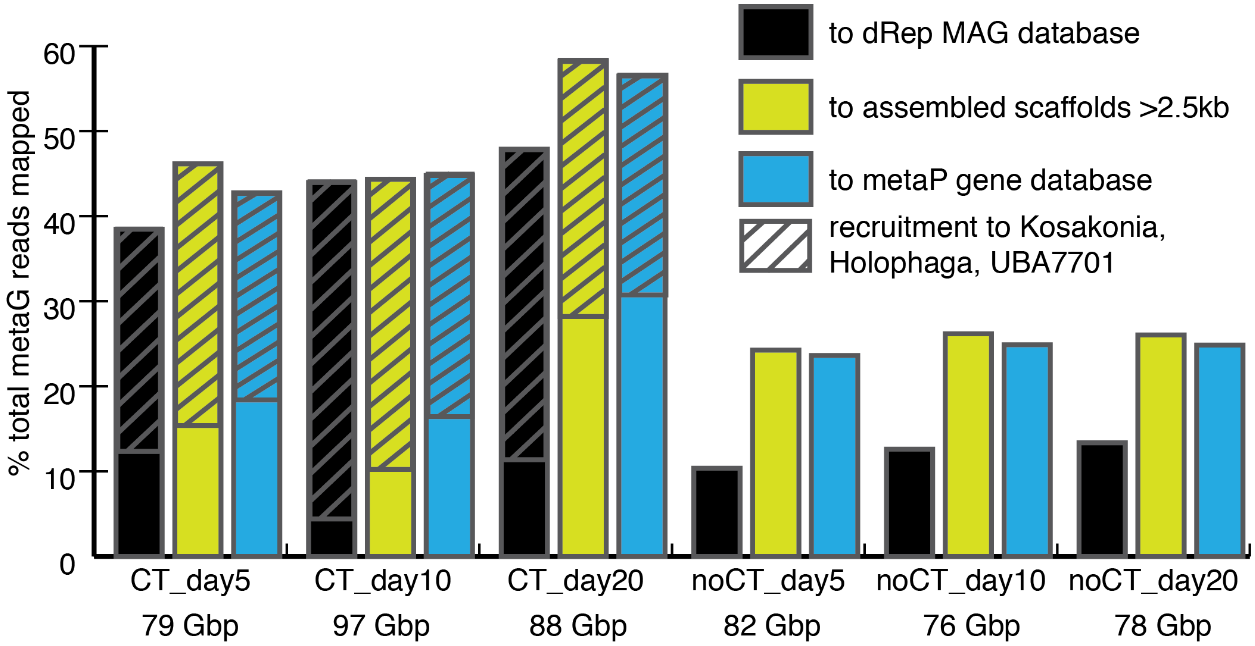

Supplementary Figure 10. Metagenomic assembly and binning equivalently captures similar proportions of the microbial communities between treatments when accounting for CT-dominant members. Relative metagenomic read recruitment to the 155 MAGs (black), to all assembled scaffolds greater than 2500 bp (green), and to the metaproteome dereplicated gene database (blue) for each metagenome. Reads were mapped at 95% identity. The fractions of reads recruited to the CT-dominant and exclusive MAGs *Kosakonia, Holophaga*, and the 3 Sporomusales UBA7701 are shown in grey stripes. The size of each metagenome is listed in gigabase pairs below sample names. These findings show that after removing the dominant lineages (grey stripes), read mapping was nearly equivalent and did not appear biased by treatment (CT versus no CT).

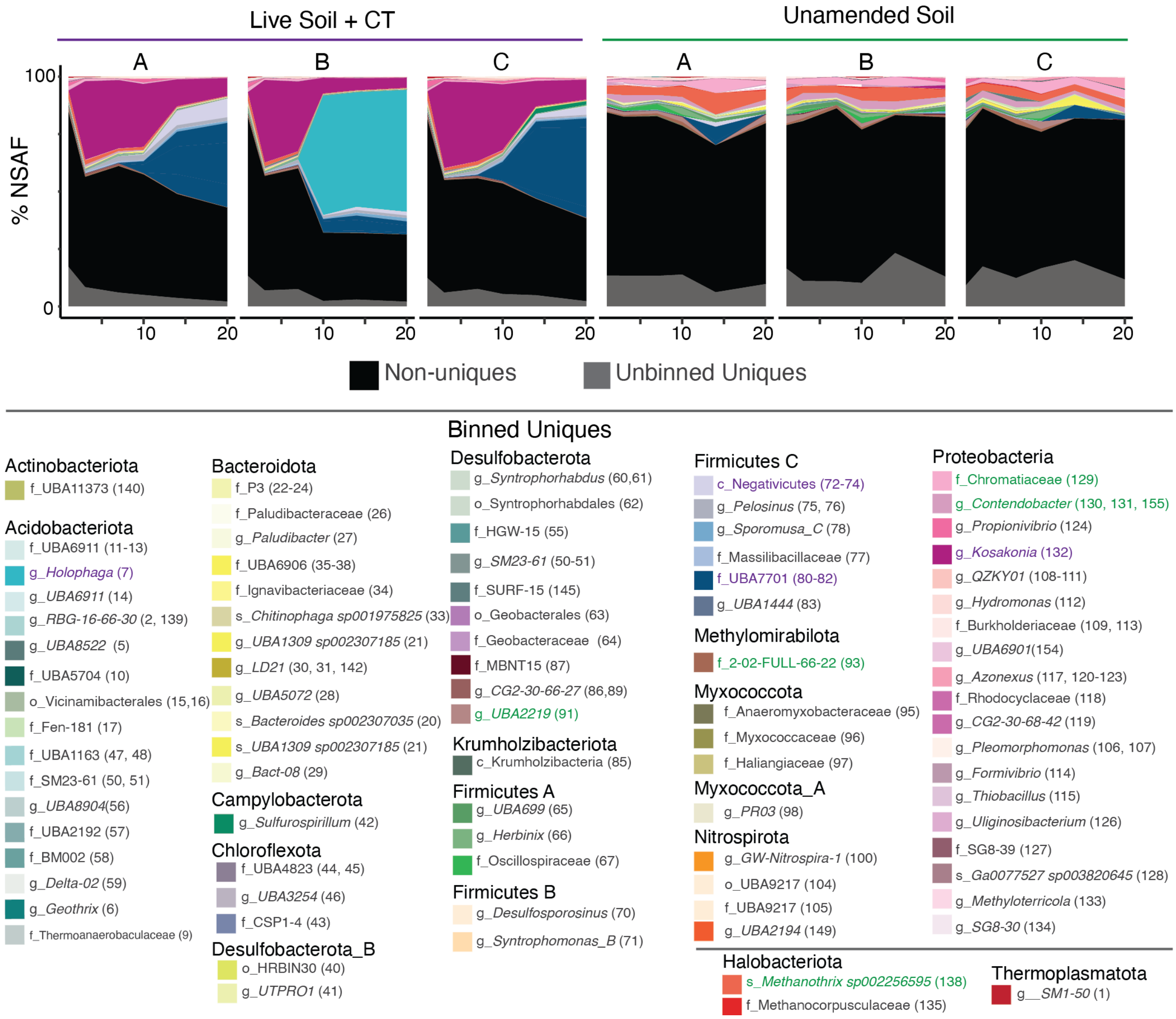

Supplementary Figure 11. Relative abundance of all classes of metaproteome hits across CT-amended and unamended samples. Metaproteomic dynamics in soil microcosms shown with the relative peptide recruitment to non-unique (black), unbinned unique (grey) and binned uniques (colored) proteins. Data are graphed for CT amended and unamended control over 20-days with replicates denoted A-C. For contributions from binned uniques alone see **Figure 5**. The color for the binned uniques (as described in **Figure 4**) is denoted by the most refined GTDB-tk assigned taxonomy, with our MAG ID number in parentheses (**Supplementary Data 2**). The names of the top 5 peptide-recruiting MAGs are colored for CT (purple) and control (green) microcosms.

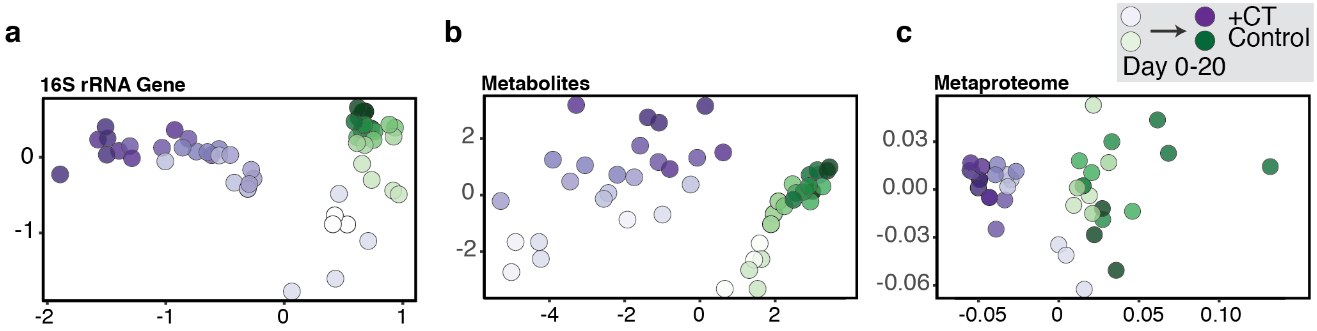

Supplementary Figure 12. CT-amended and unamended microbial communities, metabolites, and metaproteomes diverge temporally. Non-metric multidimensional scaling (NMDS) of Bray-Curtis similarity metric of **(a)** 16S rRNA gene amplicon sequencing variants (stress = 0.09), **(b)** combined NMR and LC-MS exometabolites (stress=0.08), and **(c)** metaproteome (stress=0.11). Regardless of the data type, all show statistically significant (Mrpp, $p = 0.001$ for all 3) separation by treatment and a time trajectory. Day 0 samples are white, and proceed to darker purple (CT-amended) or green (unamended).

	CTSoil_80	CTSoil_81	CTSoil_82
CTSoil_80			
CTSoil_81	72.7%, 93.01%		
CTSoil_82	71.09%, 91.27%	72.17%, 93.45%	

Supplementary Fig 13. The three Sporomusales UBA7701 MAGs are likely three different genera. Matrix of MAG Average Amino Acid identity (AAI) and ribosomal protein S3 similarity between the three Sporomusales UBA7701 MAGs. The first number in the cell is AAI, while the second value is ribosomal protein S3 amino acid identity. MAG AAI was calculated using http://enve-omics.ce.gatech.edu/aai/.

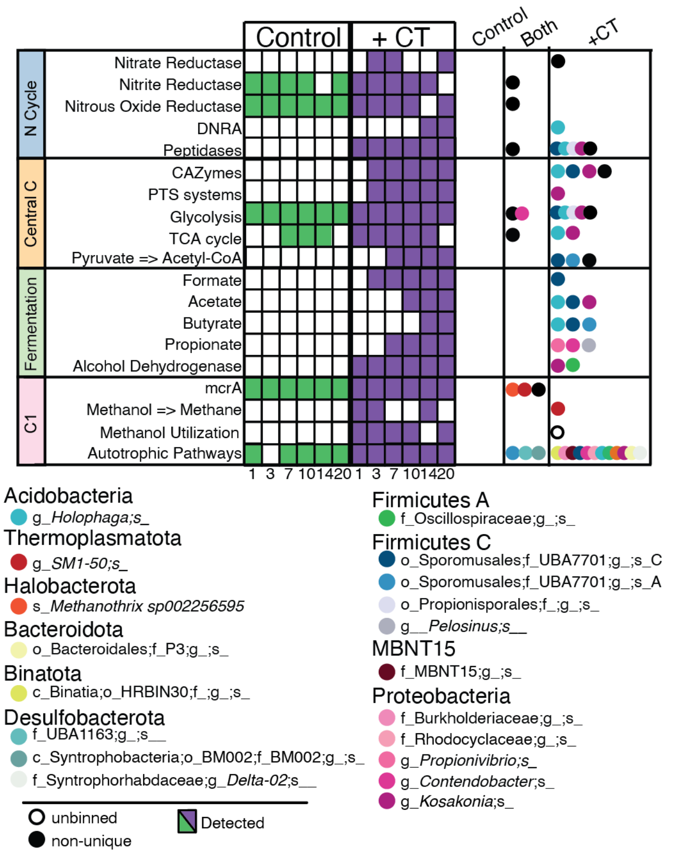

Supplementary Figure 14. Genome-resolved metaproteomic dynamics across biogeochemical processes. Processes detected in at least two of three CT (purple) or unamended control (green) microcosms at each timepoint are indicated by colored squares. The genomic origins of identified functions are indicated by circles in columns corresponding to activity in either control or CT microcosms, or activity recovered in both. Proteins that could be uniquely assigned to a MAG are colored by MAG, while hits that were unique but unable to be traced to a MAG are unfilled (unbinned uniques), while proteins that were not unique are black circles (non-uniques).

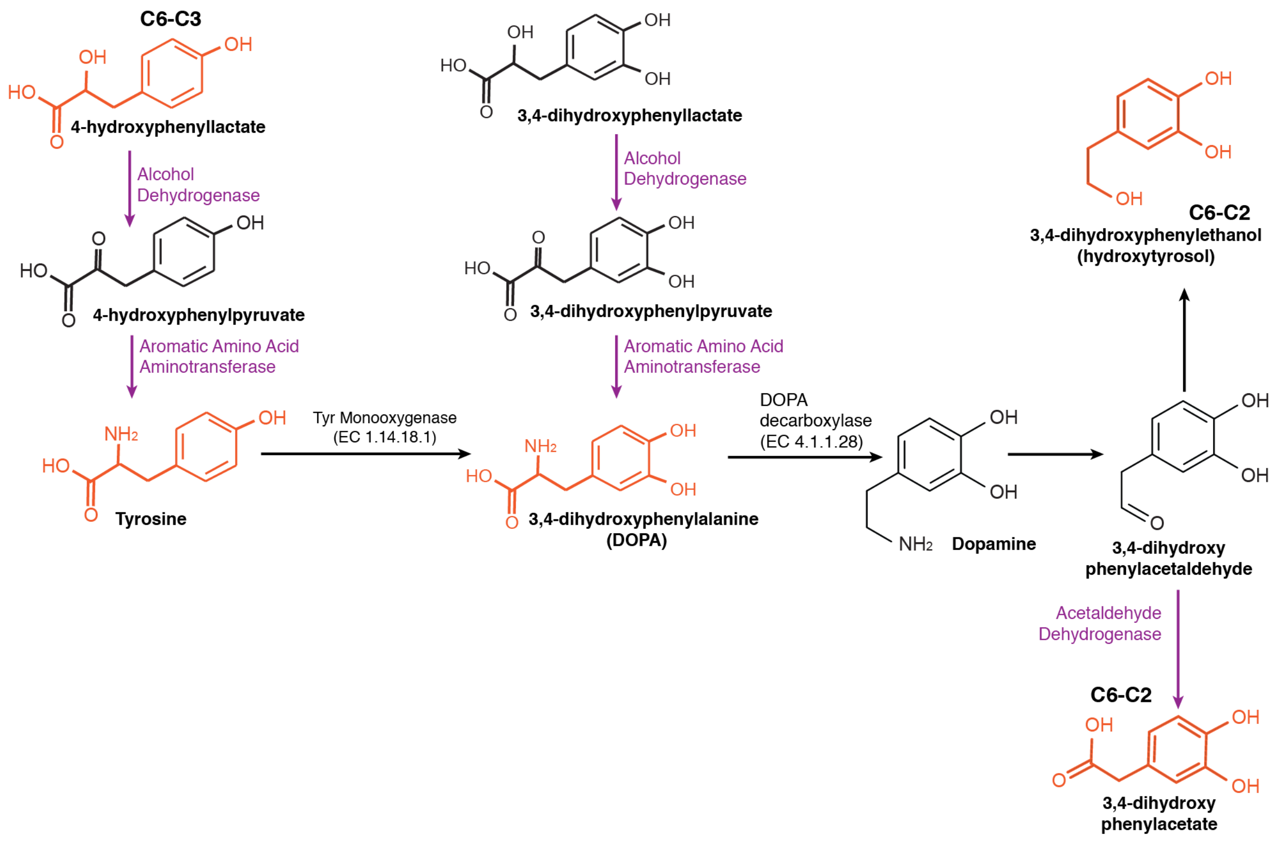

Supplementary Figure 15. Reconstructed metabolite pathway for polyphenol degradation via 3,4-dihydroxyphenylalanine (DOPA). Metabolites detected via LC-MS and NMR analyses are highlighted in orange, with putative enzymes detected via metaproteomics highlighted in purple. Known enzymes for tyrosine conversion to DOPA, and DOPA conversion to dopamine, are listed with EC numbers. In our metaproteomes, we recovered peptides corresponding to *Kosakonia* aromatic amino acid decarboxylase and alcohol dehydrogenase.

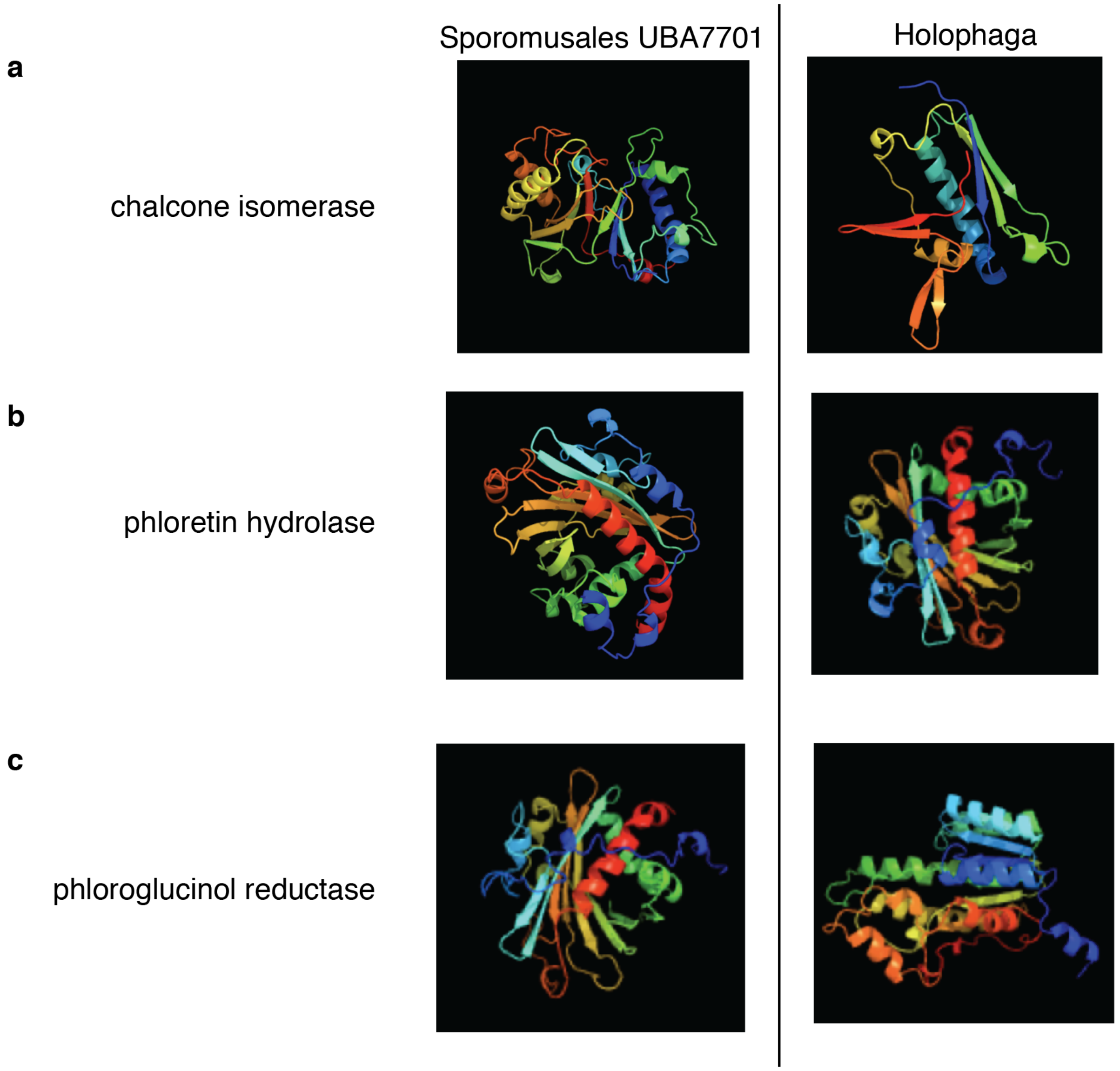

Supplementary Fig 16. Phyre2 structural models for putative flavonoid active enzymes. Structural models are shown for (**a**) chalcone isomerase, (**b**) phloretin hydrolase, and (**c**) phloroglucinol reductase. Sporomusales UBA7701 enzymes are shown on the left (chalcone isomerase and phloretin hydrolase = CTSoil_81, phloroglucinol reductase = CTSoil_82), while *Holophaga* models are shown on the right. N-terminal residues are red, and C-terminal residues are blue. Phyre2 structural model information in **Supplementary Data 3,** and gene sequences in **Supplementary Data 6**.

ASV	RefSoil Subject	%id	length	e-value	Lowest SILVA ASV taxonomy level
1	SVEN_RS37165	100	136	9.18E-65	Streptomyces
2	SVEN_RS37165	100	136	9.18E-65	Streptomyces
3	SVEN_RS37165	100	136	9.18E-65	Streptomyces
4	SVEN_RS37165	100	136	9.18E-65	Streptomyces
5	SVEN_RS37165	99.265	136	4.27E-63	*Bacillus*
6	SVEN_RS37165	97.794	136	9.24E-60	Streptomyces
7	SVEN_RS37165	97.059	136	4.30E-58	Streptomyces
8	SVEN_RS37165	97.059	136	4.30E-58	*Bacillus*
9	BSN5_RS21230	100	74	2.68E-30	Oceanobacillus;uncultured bacterium
10	BSN5_RS21230	97.297	74	5.80E-27	Streptomyces
11	BSN5_RS21230	97.297	74	5.80E-27	Streptomyces

Supplementary Table 1. Positive hits from of ASVs BLAST to RefSoil database.

	Components	Mg/L in ddiH_2O
Vitamin Mix	d-Biotin	2
	Folic Acid	2
	Pyridoxine HCl	10
	Riboflavin	5
	Thiamine	5
	Nicotinic Acid	5
	Pantothenic Acid	5
	Vitamin B12	0.1
	p-Amino benzoic Acid	5
	D,L-6,8-thiotic Acid	5
Mineral Mix	NTA Disodium Salt	1.5
	$MgSO_4$ -7H_2O	3
	$MnSO_4$ -H_2O	0.5
	NaCl	1.0
	$FeSO_4$ -7H_2O	0.1
	$CaCl_2$ -2H_2O	0.1
	$CoCl_2$ -6H_2O	0.1
	ZnCl	0.13
	$CuSO_4$- 5H_2O	0.01
	$AlK(SO_4)_2$- 12H_2O	0.01
	Boric Acid	0.01
	Na_2MoO_4- 2H_2O	0.025
	$NiCl_2$ -6H_2O	0.024
	Na_2WO_4 -2H_2O	0.025
	Na_2SeO_4	0.02

Supplementary Table 2. DL-Vitamins and Minerals in Media according to Lovley *et al.*[3]

Primer	Sequence
515F	5′-GTGCCAGCMGCCGCGGTAA-3′
806R	5′-GGACTACHVGGGTWTCTAAT-3′

Supplementary Table 3. Sequences of the Earth Microbiome Project primers used for amplicon sequencing.

Supplementary Note 1. Sterilized Soils

The soil slurry used to inoculate samples was autoclaved three consecutive times on liquid-20 cycles. Unlike the biotic samples, there was no visible biomass pellet following centrifugation of the 5mL of this sample at day 20. We attempted to extract DNA from these samples at day 20, and failed to obtain Qubit-HS detectable DNA from 5mL of the of the sample. We attempted to obtain Illumina MiSeq 16S rRNA amplicon sequencing from this sample, and we were unable to obtain any amplicons.

Supplementary Note 2. Comparison of CT degradation metabolites between soils and guts.

Many of the metabolites we detected in soils have also been reported in studies of gut microbiome metabolism of CT. However, we did not detect any phenyl valeric acids or lactones (C_6-C_5) that are widely reported as products of flavonoid metabolism in the gut[4]. These compounds are proposed to be formed by transformation of diphenylpropanes after ring opening at position 1 of the C-ring[5], where we instead proposed CT depolymerization is followed by flavonoid transformations involving C-ring fission (position 1 and 4) and release of C_6-C_3 products such as 3-hydroxyphenylpropanoic acid (**Fig 2F**). Regardless of the exact mechanisms, our data broadly echo the proposed steps for flavonoid degradation in the gut, with chain shortening by loss of CO_2 and/or acetate to yield small molecules that could enter central metabolic pathways (**Supplementary Fig 7)**.

Supplementary Note 3. Database recruitment

Our dereplicated MAG database recruited on average 43% and 12% of metagenomic reads for CT-amended and unamended samples, respectively. However, we knew the dominant MAGs *Kosakonia, Holophaga,* and the three UBA7701 MAGs were dominant in the CT-amended samples, and nearly absent in unamended samples (**Supplementary Fig 9**). Therefore, we repeated this analysis removing these MAGs and found 9% and 12% of metagenomic reads mapped to the adjusted MAG database in CT-amended and unamended samples, respectively (**Supplementary Fig 10**, black bars), thereby confirming we did not observe bias due to preferential assembly and binning with a treatment.

To assess overall metagenome assembly, we compared metagenomic read mapping to assembled scaffolds >2500 bp. We found on average 50% and 25% of reads were mapped to assembled scaffolds >2500 bp for CT-amended and unamended samples, respectively. Again, removing scaffolds from the dominant MAGs, we found 18% and 25% of reads were mapped to assembled scaffolds >2500 bp for CT-amended and unamended samples, respectively (**Supplementary Fig 10**, green bars). Therefore, while we had very dominant members in our CT amended microcosms, when we quantified read recruitment beyond those genomes we recovered similar proportions of the microbial community at the MAG and assembly levels.

To ensure we had equal representation of genes from our microbial communities in our metaproteome gene database, we again mapped our metagenomic reads to this dereplicated gene database. We found on average 40% and 22% of our reads mapped

to the database for CT-amended and unamended samples, respectively. When we removed genes derived from the five CT-dominant MAGs from this database, we saw 21% and 22% of metagenomic reads mapped from CT-amended and unamended samples, respectively (**Supplementary Fig 10**, blue bars). This again reinforced that our database captured the same proportion of the microbial communities between treatments.

Supplementary Note 4. Nitrogen Cycling

While we failed to find evidence that tannins inhibited carbon degradation, we did observe lower levels of protein-like compounds by FTICR-MS in live CT-amended reactors (**Fig 7**), which could be the result of tannin-bound organic nitrogen as has been previously suggested[6]. Furthermore, while we observed peptidases in both treatments, we detected expression of 15 peptidase-classes by 9 members under CT treatment, and just one class in the control using our unique (binned and unbinned) data. This increased peptidase diversity could reflect nitrogen limitation under CT exposure, demonstrating a shared response between our anoxic soils and previously reported aerobic soils[6]. However, we did not observe differences in the expression of denitrification enzymes (nitrate reductase, nitrite reductase, and nitrous oxide reductase) between treatments (**Supplementary Fig 14**), suggesting tannins may not repress the entire nitrogen cycle in anoxic soils.

Supplementary Note 5. HPLC and NMR Methods for characterizing purified CT

The separation used an Agilent 1100 quaternary pump system paired with an autosampler and a DAD detector collecting data at 220 nm. The column was a ThermoFisher Hypersil Gold C8, 160 x 4.6 mm column with 3 micron packing. The sample injection volume was typically 10 uL. The gradient program was controlled with ChemStation Rev. A.09.03 software and employed 0.13% (v/v) trifluoroacetic acid (TFA) in nanopure water (A) and 0.10% (v/v) TFA in acetonitrile (B) at a flow rate of 0.5 mL/min for 48 min duration. The 28 min separation phase (0-3 min, isocratic at 15% B; 3-8 min, increase to 20% B; 8-10 min, increase to 30% B; 10-28 min, isocratic at 30% B) followed by a wash & re-equilibration phase (28-32 min, increase to 70% B; 37-40 min, decrease to 15% B and hold isocratic at 15% for 8 min before the next injection).

^{1}H-^{13}C HSQC NMR spectra were recorded at 27°C on a BrukerBiospin DMX-500 (1H 500.13 MHz, 13C 125.76 MHz) instrument equipped with TopSpin 3.5 software and a cryogenically cooled 5 mm TXI 1H/13C/15N gradient probe in inverse geometry. Spectra were recorded in DMSO-d6 and were referenced to the residual signals of DMSO-d6 (2.49 ppm for 1H and 39.5 ppm for 13C spectra). For ^{1}H-^{13}C HSQC experiments, spectra were obtained using between 200 and 620 scans (depending on sample size and instrument availability) obtained using the standard Bruker pulse program (hsqcetgpsisp.2) with the following parameters: Acquisition: TD 1024 (F2), 256 (F1); SW 16.0 ppm (F2), 165 ppm (F1); O1 2350.61 Hz; O2 9431.83 Hz; D1 = 1.50 s; CNST2 = 145. Acquisition time: F2 channel, 64 ms, F1 channel 6.17 ms. Processing: SI =1024 (F2, F1), WDW = QSINE, LB = 1.00 Hz (F2), 0.30 Hz (F1); PH_mod = pk; Baseline correction ABSG = 5 (F2, F1), BCFW = 1.00 ppm, BC_mod = quad (F2), no (F1); Linear

prediction = no (F2), LPfr (F1). Sample sizes used for these spectra ranged from 5-10 mg providing NMR sample solutions with concentrations of 10-20 mg/mL.

Supplementary Note 6. Additional FTICR-MS methods

A 9.4T Tesla Bruker SolariX FTICR spectrometer located at the University of Arizona, was used to collect high resolution mass spectra of the supernatant samples from reactors (microcosms) by direct injection in negative ion mode. Samples were first desalted and concentrated using solid phase concentration (SPE) according to Dittmar et al. 2008[7]. Briefly, SPE cartridges (PPL, 300 mg, Varian Mega Bond Elut, Varian Inc., Palo Alto, CA, USA) were rinsed with one cartridge volume (3 mL) of methanol (p.a.) immediately before use. Three milliliters of each of the supernatant samples was acidified with hydrochloric acid (p.a.) to pH 2 and pumped through the SPE cartridge, at a flow rate of <50 mL/min. Before elution of DOM with methanol, the cartridges were rinsed with at least two cartridge volumes of 0.01 M HCl for complete removal of salts. Sorbents were then dried under a stream of N2 and DOM then eluted with 1 mL of methanol at a flow rate of <10 mL/min. A standard Bruker ESI source was used to generate negatively charged molecular ions and then eluted DOM samples were introduced directly to the ESI source. The instrument was externally calibrated weekly to a mass accuracy of <0.1 ppm using a tuning solution from Agilent, which contains the following compounds: $C_2F_3O_2$, $C_6HF_9N_3O$, $C_{12}HF_{21}N_3O$, $C_{20}H_{18}F_{27}N_3O_8P_3$, and $C_{26}H_{18}F_{39}N_3O_8P_3$ with an *m/z* ranging between 112 and 1333. The instrument settings were optimized by tuning on a Suwannee River Fulvic Acid (SRFA) standard. Blanks (HPLC grade MeOH) were ran at the

beginning and the end of the day to monitor potential carry over from one sample to another and the instrument was flushed between samples using a mixture of water and methanol. The ion accumulation time (IAT) was varied to account for differences in C concentration between samples. Three hundred individual scans were averaged for each sample and internally calibrated using organic matter and (epi)catechin CT oligomers homologous series separated by 14 Da (-CH_2 groups). The mass measurement accuracy was <1 ppm across a broad *m/z* range (i.e. $100 < m/z < 1000$). The mass resolution was 350,000 at 339.112 Da.

The observed spectra contained a mixture of singly and doubly charged ions (inferred from KMD analysis), and some remaining salt clusters and background noise peaks that were deleted manually. We applied Kendrick Mass Defect analysis using -CH_2 as the base unit to data from all samples. In plotting this data (**Supplementary Fig 3B**), there were clear regions that were mainly composed of points from CT-amended samples (**Supplementary Fig 3B**, red ovals). We used this to extract peaks that likely corresponded to the different oligomers of the CT polymer and derived compounds.

To verify the presence of doubly and singly charged (epi)catechin CT oligomers within our samples, we used a combination of Compass Isotope Pattern calculation and isotopic fine structure (IFS) simulation and evaluation using Bruker Data Analysis software (**Supplementary Fig 4**). In mass spectrometry, isotope profiles are displayed dependent on the effective resolution of the instrument to resolve the isotope variants. In this sense, FTICR-MS differs from lower resolution instruments since its resolving power is usually high enough to baseline separate the isotope profile of intact and multiple

charged ions. Thus, IFS analysis is useful here for identification of (epi)catechin CT oligomers since (1) IFS is an exact fingerprint for every possible molecular configuration and (2) the high resolving power of the FTICR-MS.

Kendrick mass defect analysis[8] was then used to compare the fate of (epi)catechin CT oligomers over time in both biologically active and inactive (autoclaved soil) reactors amended with CT. Kendrick plots are generated by plotting nominal mass as a function of Kendrick mass defect, most commonly based on a methylene (CH_2) subunit, although other subunits can be used (e.g., carboxyl), in a single 2D display. Thus, compounds whose elemental composition differs by a number of base units only possess the same KMD value and line up horizontally in the associated KMD plot. Here we used a modified version of KMD commonly used for polymer ions, proposed by Sato et al[1,9]. where the repeating unit of a polymer backbone (here (epi)catechin) was used as the base unit by arbitrarily setting its mass at the nearest integer and other masses are re-calculated based on that. To walk through the orientation of peaks corresponding to CT-oligomers within a Kendrick plot based on mass, charge, and isotopic composition, see **Supplementary Fig 4.**

Putative chemical formulas of the remaining singly charged ions and in-situ soil metabolites were assigned using Formularity software[10]. First, to further reduce cumulative errors, all sample peak lists for the entire dataset were aligned to eliminate possible mass shifts that would impact formula assignment. Chemical formulas were assigned based on the following criteria: $S/N > 7$, and mass measurement error < 1 ppm, taking into consideration the presence of C, H, O, N, S and P and excluding other

elements. Peaks with large mass ratios (*m/z* values > 500 Da) often have multiple possible candidate formulas. These peaks were assigned formulas through propagation of CH_2, O, and H_2 homologous series. Additionally, to ensure consistent choice of molecular formula when multiple formula candidates are found the following rules were implemented: the formula with the lowest error with the lowest number of heteroatoms was consistently picked and the assignment of one phosphorus atom required the presence of at least four oxygen atoms. Biochemical compound classes were reported as relative abundance values based on counts of C, H, and O for the following H:C and O:C ranges only : lipid-like ($0 < O:C \leq 0.3$ and $1.5 \leq H:C \leq 2.5$), unsaturated hydrocarbons-like ($0 \leq O:C \leq 0.125$ and $0.8 \leq H:C < 2.5$), protein-like ($0.3 < O:C \leq 0.55$ and $1.5 \leq H:C \leq 2.3$), carbohydrates-like ($0.55 < O:C \leq 0.7$ and $1.5 \leq H:C \leq 2.2$), lignin-like ($0.125 < O:C \leq 0.65$, $0.8 \leq H:C < 1.5$), tannin-like ($0.65 < O:C \leq 1.1$, $0.8 \leq H:C \leq 1.5$), and condensed hydrocarbon-like ($0 \leq O:C \leq 0.95$, $0.2 \leq H:C < 0.8$)[11]. To identify "polyphenol-like" compounds, we grouped lignin-like and tannin-like compounds (**Supplementary Fig 1, Fig 7**).

Supplementary Note 7. Additional LC-MS methods

Metabolites were extracted into ethyl acetate from filtered supernatant samples that were acidified with HCl. Both the aqueous and organic phases were analyzed by by liquid chromatography - tandem mass spectrometry (LC-MS/MS). Sample aliquots were stored frozen at -80˚C until extraction. Aliquots were thawed, and filtered through 0.2 um cellulose acetate to yield 1 mL filtrates that were vortexed, and then acidified by addition

of 200uL of 1M HCl. The acidified samples were then extracted 3 times with addition of 200uL of water-saturated ethyl acetate, each time followed by vortexing, sonication for 10 minutes in a water bath and collection of the ethyl acetate layer (ethyl acetate layers from the 3 extraction steps were combined). At the end the water layer was also collected. The ethyl acetate extracts were dried at room temperature in a speed vacuum concentrator; the aqueous layer was frozen at -80C and then dried by lyophilization. Dried extracts of both types were resuspended in 50uL of 80% methanol containing 1ug/mL 2-Amino-3-bromo-5-methylbenzoic acid (Sigma) as internal standard. Resuspended samples were vortexed, centrifuged (2070 RCF for 5 minutes at room temperature), then filtered via 0.22um pvdf microcentrifuge filtration tubes. Filtrates were then analyzed by LC-MS/MS using an Agilent 1290 UHPLC system connected to a Thermo Q Exactive Hybrid Quadrupole-Orbitrap Mass Spectrometer equipped with a Heated Electrospray Ionization (HESI-II) source probe. The filtrates from the ethyl acetate extraction were chromatographically separated on a ZORBAX RRHD Eclipse Plus C18, 95Å, 2.1 x 50 mm, 1.8 μm column (Agilent) and filtrates from the aqueous layer were chromatographically separated on an InfinityLab Poroshell 120 HILIC-Z, 2.1 × 150 mm, 2.7 μm (Agilent). Separation, ionization, fragmentation and data acquisition parameters are specified in **Supplementary Data 1**. Briefly, metabolites were separated by gradient elution followed by MS1 and data dependent (top 2 most abundant MS1 ions not previously fragmented in last 7 seconds) MS2 collection; targeted data analysis was performed by comparison of sample peaks to a library of analytical standards analyzed under the same conditions. Three parameters were compared: matching m/z, retention

time and fragmentation spectra using Metabolite Atlas (https://github.com/biorack/metatlas)[12,13]. Identification and standard reference comparison details are provided in **Supplementary Data 1**. Data are available for download at the JGI Joint Genome Portal under ID 1281268. To determine significantly discriminating LC-MS exometabolites, we used limma[14] in R on log2-transformed data to compare metabolites in live and autoclaved treatments at each timepoint.

References

1. Fouquet, T. N. J. *et al.* On the Kendrick Mass Defect Plots of Multiply Charged Polymer Ions: Splits, Misalignments, and How to Correct Them. *J. Am. Soc. Mass Spectrom.* **29**, 1611–1626 (2018).
2. Reeves, S. G. *et al.* Proanthocyanidin Structural Details Revealed by Ultrahigh Resolution FT-ICR MALDI-Mass Spectrometry, 1 H– 13 C HSQC NMR, and Thiolysis-HPLC–DAD. *J. Agric. Food Chem.* (2020). doi:10.1021/acs.jafc.0c04877
3. Lovley, D. R. & Phillips, E. J. P. Novel mode of microbial energy metabolism: organic carbon oxidation coupled to dissimilatory reduction of iron or manganese. *Appl. Environ. Microbiol.* **54**, 1472–1480 (1988).
4. Del Rio, D. *et al.* Dietary (poly)phenolics in human health: Structures, bioavailability, and evidence of protective effects against chronic diseases. *Antioxidants and Redox Signaling* **18**, 1818–1892 (2013).
5. Sánchez-Patán, F. *et al.* Capability of lactobacillus plantarum IFPL935 to catabolize flavan-3-ol compounds and complex phenolic extracts. *J. Agric. Food*

Chem. **60**, 7142–7151 (2012).

6. Fierer, N., Schimel, J. P., Cates, R. G. & Zou, J. Influence of balsam poplar tannin fractions on carbon and nitrogen dynamics in Alaskan taiga floodplain soils. *Soil Biol. Biochem.* **33**, 1827–1839 (2001).

7. Dittmar, T., Koch, B., Hertkorn, N. & Kattner, G. A simple and efficient method for the solid-phase extraction of dissolved organic matter (SPE-DOM) from seawater. *Limnol. Oceanogr. Methods* **6**, 230–235 (2008).

8. Hughey, C. A., Hendrickson, C. L., Rodgers, R. P., Marshall, A. G. & Qian, K. Kendrick mass defect spectrum: A compact visual analysis for ultrahigh-resolution broadband mass spectra. *Anal. Chem.* **73**, 4676–81 (2001).

9. Sato, H., Nakamura, S., Teramoto, K. & Sato, T. Structural characterization of polymers by MALDI spiral-TOF mass spectrometry combined with kendrick mass defect analysis. *J. Am. Soc. Mass Spectrom.* **25**, 1346–1355 (2014).

10. Tolić, N. *et al.* Formularity: Software for Automated Formula Assignment of Natural and Other Organic Matter from Ultrahigh-Resolution Mass Spectra. *Anal. Chem.* **89**, 12659–12665 (2017).

11. Tfaily, M. M. *et al.* Sequential extraction protocol for organic matter from soils and sediments using high resolution mass spectrometry. *Anal. Chim. Acta* **972**, 54–61 (2017).

12. Yao, Y. *et al.* Analysis of metabolomics datasets with high-performance computing and metabolite atlases. *Metabolites* **5**, 431–442 (2015).

13. Bowen, B. P. & Northen, T. R. Dealing with the unknown: Metabolomics and

metabolite atlases. *J. Am. Soc. Mass Spectrom.* **21**, 1471–1476 (2010).

14. Smyth, G. K. limma: Linear Models for Microarray Data. in *Bioinformatics and Computational Biology Solutions Using R and Bioconductor* 397–420 (Springer-Verlag, 2005). doi:10.1007/0-387-29362-0_23
