# Supplementary material for: Decrypting bacterial polyphenol metabolism in an anoxic wetland soil
Source: Nat Commun. 2021 Apr 29;12:2466. doi: 10.1038/s41467-021-22765-1 (PMC8084988; doi:10.1038/s41467-021-22765-1)
Supplement: Supplementary file 2 — Description of Additional Supplementary Files [file 41467_2021_22765_MOESM2_ESM.docx]

Description of Additional Supplementary Files

File Name: Supplementary Data 1

Description: Metabolite identification data for LC-MS and NMR, including specifics of LC-MS methodology and LIMMA statistical significance.

File Name: Supplementary Data 2

Description: (xlsx) Information on metagenomes, assemblies, MAGs, and 16S rRNA gene reads, including accession numbers.

File Name: Supplementary Data 3

Description: (xlsx) Information on metaproteomes including table of MAG peptide recruitment by sample, peptide recruitment/functional annotation for detected proteins, and enzyme information for flavonoid enzymes.

File Name: Supplementary Data 3

Description: (xlsx) Information on metaproteomes including table of MAG peptide recruitment by sample, peptide recruitment/functional annotation for detected proteins, and enzyme information for flavonoid enzymes.

File Name: Supplementary Data 4

Description: (xlsx) Feature table derived from 16S rRNA gene analyses.

File Name: Supplementary Data 5

Description: (amino acid FASTA) Flavonoid enzymes queried against this dataset, with accession numbers in FASTA header.

File Name: Supplementary Data 6

Description: (amino acid FASTA) Amino acid sequences of Flavonoid enzyme encoding genes CHI, FCR, PHY, and PGR, and *Kosakonia* KatG and AA6.

File Name: Supplementary Data 7

Description: (xlsx) FTICR-MS data for all peaks across all samples, including formula assignment if applicable and Kendrick Mass defect analysis.
