# Supplementary material for: Decrypting bacterial polyphenol metabolism in an anoxic wetland soil
Source: Nat Commun. 2021 Apr 29;12:2466. doi: 10.1038/s41467-021-22765-1 (PMC8084988; doi:10.1038/s41467-021-22765-1)
Supplement: Supplementary file 10 — Reporting Summary [file 41467_2021_22765_MOESM10_ESM.pdf]

nature research

Corresponding author(s): Kelly Wrighton

Last updated by author(s): Mar 1, 2021

Reporting Summary

Nature Research wishes to improve the reproducibility of the work that we publish. This form provides structure for consistency and transparency in reporting. For further information on Nature Research policies, see our Editorial Policies and the Editorial Policy Checklist.

Statistics

For all statistical analyses, confirm that the following items are present in the figure legend, table legend, main text, or Methods section.

n/a	Confirmed	
☐	☒	The exact sample size (*n*) for each experimental group/condition, given as a discrete number and unit of measurement
☐	☒	A statement on whether measurements were taken from distinct samples or whether the same sample was measured repeatedly
☐	☒	The statistical test(s) used AND whether they are one- or two-sided<br>*Only common tests should be described solely by name; describe more complex techniques in the Methods section.*
☒	☐	A description of all covariates tested
☒	☐	A description of any assumptions or corrections, such as tests of normality and adjustment for multiple comparisons
☐	☒	A full description of the statistical parameters including central tendency (e.g. means) or other basic estimates (e.g. regression coefficient) AND variation (e.g. standard deviation) or associated estimates of uncertainty (e.g. confidence intervals)
☐	☒	For null hypothesis testing, the test statistic (e.g. *F*, *t*, *r*) with confidence intervals, effect sizes, degrees of freedom and *P* value noted<br>*Give P values as exact values whenever suitable.*
☒	☐	For Bayesian analysis, information on the choice of priors and Markov chain Monte Carlo settings
☒	☐	For hierarchical and complex designs, identification of the appropriate level for tests and full reporting of outcomes
☒	☐	Estimates of effect sizes (e.g. Cohen's *d*, Pearson's *r*), indicating how they were calculated

Our web collection on statistics for biologists contains articles on many of the points above.

Software and code

Policy information about availability of computer code

Data collection	Methane data was collected using Shimadzu (GC-2014) gas chromatograph platform. 16S amplicon sequencing data was collected on the Illumina MiSeq platform. Metagenomic sequencing data was collected using the Illumina NovaSeq 6000 platform. Metaproteomic data was collected using a commercial Thermo Scientific Orbitrap Lumos platform. FT-ICRMS data was collected using a commercial Bruker platform. LC-MS metabolite data was collected using a commercial Agilent platform. NMR metabolite data was collected using a commercial Varian platform.
Data analysis	The following software was used to proccess 16S amplicon sequencing data: QIIME2 (2017.10), SILVA classifier (silva132.250).<br><br>The following software was used to process metagenomic sequencing data: Sickle (v 1.33), IDBA-UD (v1.1.0), MetaBAT2 (v2.12.1), AMPHORA2 (https://github.com/martinwu/AMPHORA2/branches), checkM (v1.1.2), dRep (v2.6.2), GTDB-tk (v1.3.0) R05-RS95, DRAM (v1.0), bbmap (v38.70), samtools (v1.9), coverM (v0.3.2).<br><br>The following software was used to analyze potential proteins: PHYRE2 (http://www.sbg.bio.ic.ac.uk/~phyre2/html/page.cgi?id=index).<br><br>The following software was used to analyze metaproteome data: MS-GF+ (no version noted, https://omics.pnl.gov/software/ms-gf).<br><br>The following software was used to analyze FT-ICRMS data: Formularity (v1.0.0).<br><br>The following software was used to analyze LC-MS data: Metabolite Atlas (https://github.com/biorack/metatlas).<br><br>The following software was used to analyze the NMR data: Chenomx NMR Suite (v8.3), MestReNova (v14.0).

The following software was used to analyze the data in R: limma (v3.42.2).

For manuscripts utilizing custom algorithms or software that are central to the research but not yet described in published literature, software must be made available to editors and reviewers. We strongly encourage code deposition in a community repository (e.g. GitHub). See the Nature Research guidelines for submitting code & software for further information.

Data

Policy information about availability of data

All manuscripts must include a data availability statement. This statement should provide the following information, where applicable:

- Accession codes, unique identifiers, or web links for publicly available datasets
- A list of figures that have associated raw data
- A description of any restrictions on data availability

The MAGs, assemblies, and reads resolved from the microcosm dataset reported in this paper have been deposited in National Center for Biotechnology Information BioProject (accession number PRJNA638681; for biosample accession numbers see Supplementary Data 1). The mass spectrometry proteomics data have been deposited to the ProteomeXchange Consortium via the PRIDE partner repository with the dataset identifier PXD019911 and 10.6019/PXD019911. LC-MS data are available for download at the JGI Joint Genome Portal under ID 1281268. FT-ICRMS and NMR data have been deposited in the following archive: doi:10.5281/zenodo.4552584.

All data underlying figures presented are provided as supplementary data with the paper.
External databases used in this study: RefSoil (https://figshare.com/articles/dataset/RefSoil_Database/4362812).

Field-specific reporting

Please select the one below that is the best fit for your research. If you are not sure, read the appropriate sections before making your selection.

☐ Life sciences ☐ Behavioural & social sciences ☒ Ecological, evolutionary & environmental sciences

For a reference copy of the document with all sections, see nature.com/documents/nr-reporting-summary-flat.pdf

Ecological, evolutionary & environmental sciences study design

All studies must disclose on these points even when the disclosure is negative.

Study description	Anoxic soil microcosms were constructed, and amended with condensed tannin (n=3) or without (n=3). We had an autoclaved soil control (n=1) that was also amended with condensed tannin. The microcosms were sampled over 20-days for various multi-omic analyses.
Research sample	The soil used in this study was taken from Old Woman Creek, Ohio from a plant-covered surface soil because this soil is well-studied, and known to be exposed to fluctuating redox and polyphenols. The soil sample was meant to represent the broader wetland soil microbial community.<br><br>Over the course of 20-days, we periodically sampled these microcosms for 16S amplicon sequencing, metagenomes, metaproteomes, and metabolomic analyses. These samples represent the various sampled parameters of the microbial communities at individual timepoints.
Sampling strategy	We designed our experiment to have triplicate CT-amended and unamended microcosms, as well as a single autoclaved soil replicate. We sampled these microcosms over the course of 20-days to capture the microbial community response based on the various sampled community parameters. We chose this experimental design to ensure biological replication across the sampling scheme, and the choice of a single autoclaved reactor was made based on limited amounts of CT to set up the experiment. This is still robust because we have the biological replicates, and we are conservative by using the autoclaved sample to suggest that trends observed in the biological samples may have an abiotic component.<br><br>The microcosms were anoxically-sampled at the various timepoints, and samples were stored at -80C until analysis.
Data collection	B.B.M., M.A.B., and R.A.D. executed the microcosm experiment. B.B.M. anoxically sampled the microcosms at the indicated timepoints as in DOI: 10.1128/mSystems.00320-19. B.B.M. extracted DNA using the Qiagen DNeasy PowerSoil Kit.<br>A.E.H. purified and characterized the CT used in this experiment as in: doi.org/10.1021/acs.jafc.0c04877.<br><br>16S amplicon sequencing library preparation and sequencing was done at Argonne National Laboratory's Next Generation Sequencing Facility on the Illumina MiSeq. Metagenomic DNA was prepared for metagenomic sequencing using the Nextera XT Low Input-Illumina library creation kit, and was sequenced at the Department of Energy Joint Genome Institute on the Illumina NovaSeq 6000.<br><br>M.M.T. carried out the FTICR-MS data collection on a 9.4 Tesla Bruker SolariX FTICR spectrometer.<br><br>S.M.K. and T.R.N. collected LC-MS metabolite data on an Agilent 1290 UHPLC system connected to a Thermo Q Exactive Hybrid Quadrupole-Orbitrap Mass Spectrometer equipped with a Heated Electrospray Ionization (HESI-II) source probe.<br><br>C.D.N., S.O.P., and M.S.L. performed metaproteomics data collection using an Orbitrap Lumos (Thermo Scientific) connected to a

	nanoACQUITY UPLC M-Class liquid chromatography system (Waters) via in-house 30-CM x 75-uM column packed using Reprocil-pur 1.9-µm C18 particles (Dr. Maisch HPLC GmbH, Germany) and in-house built electrospray apparatus.<br><br>A.R.W. and D.W.H. carried out the NMR data collection using a Varian 600 MHz VNMRS spectrometer.
Timing and spatial scale	Field Soil used as inoculum for the microcosms was taken August 15, 2015 from OWC National Estuarine Research Reserve (41°22′N 82°30′W) as described in: doi.org/10.1038/s41467-017-01753-4. After prior research using this field soil indicated the microbial community in the soil was adapted to fluctuating redox, and exposed to polyphenols, we selected it as our inoculum. Samples were stored at -80C until the day of microcosm construction. Microcosms were constructed on June 19, 2018. Samples were taken on day 0 (June 19, 2018), day 3, day 5, day 7, day 10, day 14, and day 20 (July 9, 2018). This sampling timescale was designed after preliminary (unpublished) experiments indicated this was the timescale for CT degradation.
Data exclusions	No data was excluded from the manuscript.
Reproducibility	We had biological replicate microcosms (n=3) for each treatment. As soil microbial communities are diverse and functionally complex, we saw biological replicates showed largely reproducible community composition and function, and in metabolite dynamics. Any areas where we did not see reproducible results across the biological replicates are noted in the text, notably that one CT-amended microcosm showed Holophaga-enrichment, while the other two microcosms showed Sporomusales UBA7701 enrichment.
Randomization	Samples were taken from microcosms in random order. DNA was extracted from samples in random batches. 16S amplicon sequencing library prep was conducted in random order, and all samples sequenced in the same run. Metagenome sequencing library prep was carried out in random order, and samples sequenced in the same run. For metaproteomes, LC-MS, NMR, and FTICRMS, samples were analyzed in a random order.
Blinding	Blinding was not required for this experiment as it did not involve human subjects, but samples from soil.

Did the study involve field work? ☐ Yes ☒ No

Reporting for specific materials, systems and methods

We require information from authors about some types of materials, experimental systems and methods used in many studies. Here, indicate whether each material, system or method listed is relevant to your study. If you are not sure if a list item applies to your research, read the appropriate section before selecting a response.

Materials & experimental systems

n/a	Involved in the study
☒	☐ Antibodies
☒	☐ Eukaryotic cell lines
☒	☐ Palaeontology and archaeology
☒	☐ Animals and other organisms
☒	☐ Human research participants
☒	☐ Clinical data
☒	☐ Dual use research of concern

Methods

n/a	Involved in the study
☒	☐ ChIP-seq
☒	☐ Flow cytometry
☒	☐ MRI-based neuroimaging
